# BHLHE40, a potential immune therapy target, regulated by FGD5-AS1/miR-15a-5p in pancreatic cancer

**DOI:** 10.1038/s41598-023-43577-x

**Published:** 2023-09-29

**Authors:** Wenxin Qi, Qian Liu, Wenjun Fu, Jiaming Shi, Minmin Shi, Songqi Duan, Zhe Li, Shaohua Song, Jiao Wang, Yihao Liu

**Affiliations:** 1 School of Life Sciences, Shanghai University, Shanghai, China; 2 Department of General Surgery, Ruijin Hospital, Shanghai Jiaotong University School of Medicine, Shanghai, 200025 China; 3 College of Food Science, Sichuan Agricultural University, Yaan, China

**Keywords:** Pancreatic cancer, Tumour biomarkers, Tumour immunology

## Abstract

Pancreatic cancer, as one of the neoplasms with the highest degree of malignancy, has become a main disease of concerns in recent years. BHLHE40, a critical transcription factor for remodeling of the tumor immune microenvironment, has been described to be substantially increased in a variety of tumor-associated immune cells. Nevertheless, the pro-cancer biological functions and underlying molecular mechanisms of BHLHE40 for pancreatic cancer and its unique microenvironment are unclear. Hereby, we investigated the pro-oncogenic role of BHLHE40 in the pancreatic cancer microenvironment by bioinformatics analysis and cell biology experiments and determined that the expression of BHLHE40 was obviously elevated in pancreatic cancer tissues than in adjacent normal tissues. In parallel, Kaplan–Meier survival analysis unveiled that lower expression of BHLHE40 was strongly associated with better prognosis of patients. Receiver operating characteristic (ROC) curve analysis confirmed the accuracy of the BHLHE40-related prediction model. Subsequent, spearman correlation analysis observed that higher expression of BHLHE40 might be involved in immunosuppression of pancreatic cancer. Silencing of BHLHE40 could inhibit proliferation, invasion, and apoptosis of pancreatic cancer in vitro and in vivo, implying that BHLHE40 is expected to be a potential therapeutic target for pancreatic cancer. In addition, we explored and validated the FGD5-AS1/miR-15a-5p axis as a potential upstream regulatory mode for high expression of BHLHE40 in pancreatic cancer. In summary, our data showed that ceRNA involved in the regulation of BHLHE40 contributes to the promotion of immunosuppressive response in pancreatic and is expected to be a diagnostic marker and potential immunotherapeutic target for pancreatic cancer.

## Introduction

Pancreatic cancer is a devastating malignant tumor, and with the rapid growth of incidence and mortality, it is expected to be the second leading cause of cancer-related mortality in the United States before 2030^1,2^. The poor prognosis is due to difficulty in early diagnosis, the lesion sites are prone to liver or blood metastasis and immunosuppressive components in microenvironment^3,4^. Pancreatic cancer is most common in the United States, Europe and Australia, and pancreatic cancer involves genetics, diabetes, diet and smoking^5–7^. Remarkable progress has been made in treatment of pancreatic cancer, including the introduction of surgical techniques and medical therapies such as laparoscopic techniques and neo-adjuvant chemoradiotherapy^8,9^. However, 85% of patients present with metastatic unresectable pancreatic cancer and pancreatic cancer responds poorly to most chemotherapeutic agents^10^. Hence, it is essential to dig deeper into the molecular pathways that are contained in the process of pancreatic cancer to find novel biomarkers to improve the prognosis of pancreatic cancer patients^11,12^.

Transcripts of Long non-coding RNAs (LncRNA) specifically bind to transcription factors (TF) or form scaffold structures for transcription initiation to control disease genesis and progression, e.g. ZEB1 may emerge as a mediator of linc-ROR-induced epithelial-mesenchymal transition (EMT) to promote invasion and metastasis in pancreatic cancer^13,14^, TF-LncRNA-mediated feed-forward loop networks are involved in prognostic motifs in different cancers^15^. As a TF, BHLHE40 can be expressed in both nucleus and cytoplasm, regulating different targets in variant organs. Besides, BHLHE40 is a helix-loop-helix transcription factor that has been emerged as a key immunoregulatory factor, regulating cytokine production in T cells and the proliferation of macrophages^16–18^. As a result, it shows a discrepancy on the influence of tumorigenesis, namely, BHLHE40 overexpressed in brain, breast, and gastric tumors but downregulated in colorectal, lung^19,20^. In pancreatic cancer, the observed higher BHLHE40 levels in pancreatic cancer compared with the low levels in non-tumor tissues, and the low expression of BHLHE40 is associated with a good prognosis of pancreatic cancer^20^. BHLHE40 affect the polarization and differentiation of neutrophils. Moreover, there’s a strong relationship between BHLHE40 infiltration and prognosis^21^. It’s clear that BHLHE40 drives tumorigenesis of neutrophils. Salmon’s study reveals a key role for BHLHE40 in effective Immune checkpoint therapy (ICT) and suggests that BHLHE40 may be a predictive or prognostic biomarker of ICT efficacy and a potential therapeutic target^22–24^. Given the importance of the phenomenon of immune infiltration in tumorigenesis and progression, an in-depth exploration of the molecular mechanisms underlying the highly immunosuppressive environment of PDAC has become an urgent need for the treatment of pancreatic cancer^25,26^. Recent studies have shown that targeting crosstalk between tumour cells and tumor microenvironment (TME) holds promise as a new therapeutic intervention for pancreatic cancer and that patients with higher levels of T cell infiltration are often more sensitive to immunotherapy^27–29^.

LncRNAs have been found to have a crucial regulatory on the initiation and progression of pancreatic cancer^30,31^. Many studies have shown that LncRNAs can be used as biomarkers for diagnosis and prognosis of tumors and can be detected in plasma^32–34^. LncRNAs influence gene expression through various mechanisms that include transcriptional interference, remodeling of chromatin, splicing modulation, translating regulation by combining with ribosomes or translation factors, acting as competing endogenous RNAs for miRNAs, altering proteins localization, modulating telomere replication, and RNA interference^35–37^. Based on these functions in cellular and biochemical processes, lncRNAs have been regarded as promising biomarkers and targets of treatment^38,39^.

In our study, The Cancer Genome Atlas (TCGA), Genotype-Tissue Expression (GTEX) datasets was used to perform Kaplan–Meier (KM) survival and ROC analyses to explore BHLHE40 expression and clinical significance in several cancers. Moreover, we analyzed the correlation between BHLHE40 expression and immune activation was analyzed to show that BHLHE40 expression in pancreatic cancer was closely related to immunosuppression. Lastly, we identified the biological function of BHLHE40 and revealed that the FGD5-AS1/miR-15a-5p axis was the most probable lncRNA-related pathway in pancreatic cancer. Altogether, we demonstrated the potential function of BHLHE40 as a regulator of tumor progression and its value as a direct therapeutic target and a prognostic marker of pancreatic cancer.

## Results

### Landscape of BHLHE40 expression in pan-cancer and BHLHE40 is a significant prognostic predictor in pancreatic cancer

**Figure 1:**
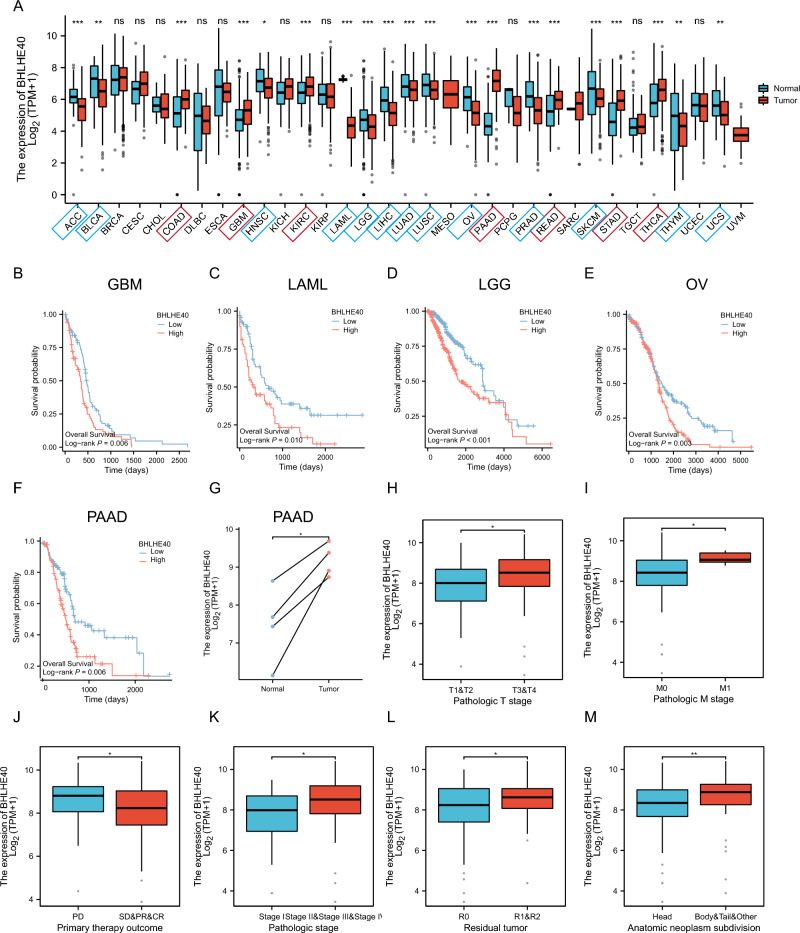
Expression pattern and prognostic value of BHLHE40 from the perspective of pan-cancer. (**A**) BHLHE40 expression levels in different tumor tissues and adjacent normal tissues from TCGA databases. (**B–F**) Prognostic analysis of BHLHE40 mRNA expression levels in glioblastoma (GBM), acute myeloid leukemia (LAML), brain lower grade glioma (LGG), ovarian serous cystadenocarcinoma (OV) and pancreatic adenocarcinoma (PAAD). (**G**) BHLHE40 mRNA expression levels in PAAD patients and normal paired sample from TCGA databases. (**H–M**) BHLHE40 mRNA expression levels in T stage (T3 and T4 stage vs. T1 and T2 stage), M stage, pathologic stage (stage II and III and IV vs. stage I), primary therapy outcomes (PD vs SD and PR and CR), Residual tumor (R1 and R2 vs. R0) and Anatomic neoplasm subdivision (Body and Tails and Others vs. Head).

**Figure 2:**
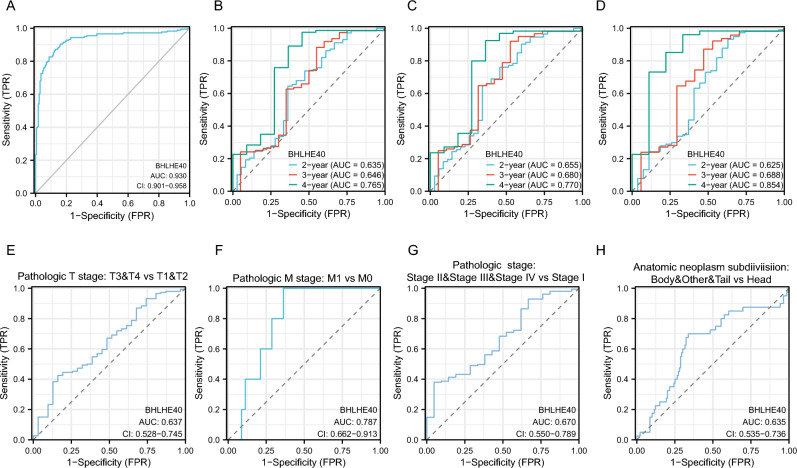
Diagnostic value of BHLHE40 for PDAC diagnosis. (**A**) Receiver operating characteristic (ROC) curves for BHLHE40 to diagnose PAAD in the TCGA database. (**B**) Time-dependent ROC curves used in the TCGA database to predict the value of BHLHE40 in diagnosing PAAD at 2-, 3-, and 4-years OS. (**C,D**) Time-dependent ROC curves for predicting 2-, 3- and 4-year DSS (**C**) and PFI (**D**) in the PAAD-TCGA database. (**E–H**) Subgroups of ROC) curves for BHLHE40, including pathologic T stage: T3 and T4 vs T1 and T2 (**E**), pathologic M stage: M1 vs M0 (**F**), Pathologic stage: stage II and III and IV vs stage I (**G**), Anatomic neoplasm subdivision: Body and Other and Tail vs Head (**H**) to diagnose PAAD in the TCGA database.

**Figure 3:**
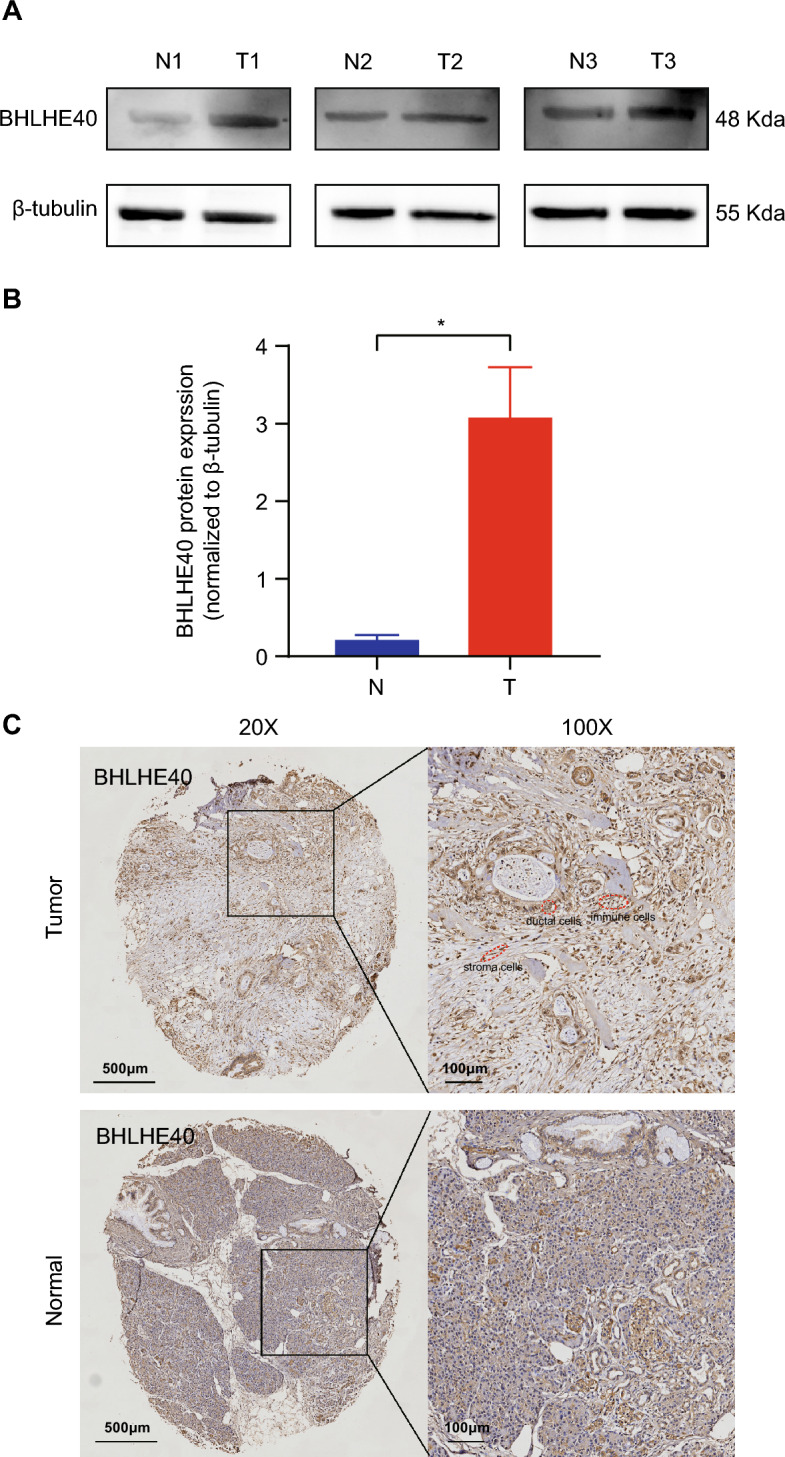
The protein level of BHLHE40 is upregulated in tumor tissues compared to normal tissues from pancreatic cancer patients. (**A,B**) WB and gray value analysis of BHLHE40 in tumor and normal tissues (n = 3). (**C**) IHC analysis of BHLHE40 in tumor and normal tissues. *P < 0.05; **P < 0.01; ***P < 0.001; ****P < 0.0001.

To investigate the expression of BHLHE40 in pan-cancer, we used TCGA and GTEX database including tumor tissues and normal tissues from 33 types of cancer to reveal that BHLHE40 were highly expressed in 7 of 33 tumor tissues compared to para-cancer tissues and were downregulated in 13 of 33 cancers (Fig. 1A). Further, Kaplan–Meier survival curves and log-rank test of these 20 types of cancers were assessed to identify that higher expression of BHLHE40 were correlated with worse prognosis in Glioblastoma multiforme (GBM), Acute Myeloid Leukemia (LAML), Brain Lower Grade Gliom (LGG), Ovarian serous cystadenocarcinoma (OV) and Pancreatic adenocarcinoma (PAAD) (Fig. 1B–F, Fig. S1A–O). Meanwhile, higher expression of BHLHE40 was significantly associated with shorter Disease Free Survival (DSS) (Fig. S2A) and Progression Free Interval (PFI) (Fig. S2B) in PDAC patients. In addition, paired tumor tissues and normal tissues in PAAD from TCGA dataset were performed that BHLHE40 was higher expressed in tumor tissues (Fig. 1G). To further assess BHLHE40 expression at different pathological stages, TCGA datasets were used to perform that the over-expressed RNA level of BHLHE40 was correlated with T stage (T3 and T4 stage vs. T1 and T2 stage) (Fig. 1H), M stage (Fig. 1I), pathologic stage (stage II and III and IV vs. stage I) (Fig. 1J), primary therapy outcomes (Progressive disease, PD vs Stable disease, SD& Partial response, PR& Complete response, CR) (Fig. 1K), Residual tumor (R1 and R2 vs. R0) (Fig. 1L) and Anatomic neoplasm subdivision (Body and Tails and Others vs. Head) (Fig. 1M), and the expression of BHLHE40 is not significantly different between groups classified based on Gender (Fig. S2C), Age (Fig. S2D), Alcohol history (Fig. S2E), History of chronic pancreatitis (Fig. S2F), Family history of cancer (Fig. S2G), History of diabetes (Fig. S2H), and Pathologic N stage (Fig. S2I). Subsequently, the ROC curve revealed that the expression of BHLHE40 had high accuracy (The Area Under Curve (AUC) 0.93, 95% confidence interval [CI] 0.901–0.958) for the diagnosis of PDAC (Fig. 2A). According to the time-dependent ROC curves, the expression of BHLHE40 performed well in predicting 2-year (C statistic, 0.635), 3-year (C statistic, 0.646) and 4-year OS (C statistic, 0.765) (Fig. 2B) in PDAC patients. The AUC values of the time-dependent ROC curves for DSS and PFI were 0.655, 0.680, 0.770, and 0.625, 0.688, and 0.854 for 2, 3, and 4 years from the PAAD-TCGA dataset (Fig. 2C,D). Furthermore, it was shown that AUC values of BHLHE40 for predicting outcomes of PDAC such as T stage (Fig. 2E), M stage (Fig. 2F), pathologic stage (Fig. 2G) and anatomic neoplasm subdivision (Fig. 2H) were high. In parallel, univariate COX and multifactorial COX analyses including BHLHE40 revealed that BHLHE40 potentially serves as a prognostic predictor for patients with pancreatic cancer as a carcinogenic factor but is dependent on clinicopathological characteristics: pathological T and N, pathological stage, primary therapy outcome, and residual tumor (Table 1). To explore the protein level of BHLHE40 in PDAC tissues, WB analysis was displayed that BHLHE40 was over-expressed in tumor tissues compared to paired-noncancerous tissues from three PDAC patients (Fig. 3A,B). IHC staining of BHLHE40 in PDAC tissues from pancreatic cancer patients showed that BHLHE40 located in the nucleus was highly expressed in tumor tissues compared to the normal tissue and its highly expressed in the ductal cells, stroma cells, immune cells (Fig. 3C). Considering all the above results, supported the prognostic value of BHLHE40 and its possible significance in the pathogenesis of PDAC.

**Table 1:** Univariate COX analysis and multivariate analysis of BHLHE40 in PAAD from TCGA datasets.

Characteristics	Total (N)	Univariate analysis		Multivariate analysis	
		Hazard ratio (95% CI)	P value	Hazard ratio (95% CI)	P value
Pathologic T stage	177		**0.045**		
T1 and T2	31	Reference		Reference	
T3	143	2.056 (1.090–3.878)	**0.026**	1.619 (0.581–4.513)	0.357
T4	3	1.091 (0.140–8.489)	0.934	1.375 (0.141–13.425)	0.784
Pathologic N stage	174		**0.002**		
N0	50	Reference		Reference	
N1	124	2.161 (1.287–3.627)	**0.004**	1.719 (0.827–3.574)	0.147
Pathologic M stage	85		0.713		
M0	80	Reference			
M1	5	0.773 (0.185–3.227)	0.724		
Pathologic stage	176		**0.018**		
Stage I	21	Reference		Reference	
Stage II and stage III and stage IV	155	2.309 (1.059–5.033)	**0.035**	0.694 (0.165–2.918)	0.618
Primary therapy outcome	140		** < 0.001**		
PD	50	Reference		Reference	
SD and PR and CR	90	0.403 (0.255–0.637)	** < 0.001**	0.530 (0.324–0.868)	**0.012**
Residual tumor	165		**0.028**		
R0	107	Reference		Reference	
R1 and R2	58	1.650 (1.064–2.558)	**0.025**	1.426 (0.863–2.356)	0.166
BHLHE40	179		**0.006**		
Low	89	Reference		Reference	
High	90	1.787 (1.177–2.712)	**0.006**	1.287 (0.786–2.106)	0.316

Significant values are in bold.

### Intrinsic association between BHLHE40 expression and clinicopathological variables in pancreatic cancer

**Figure 4:**
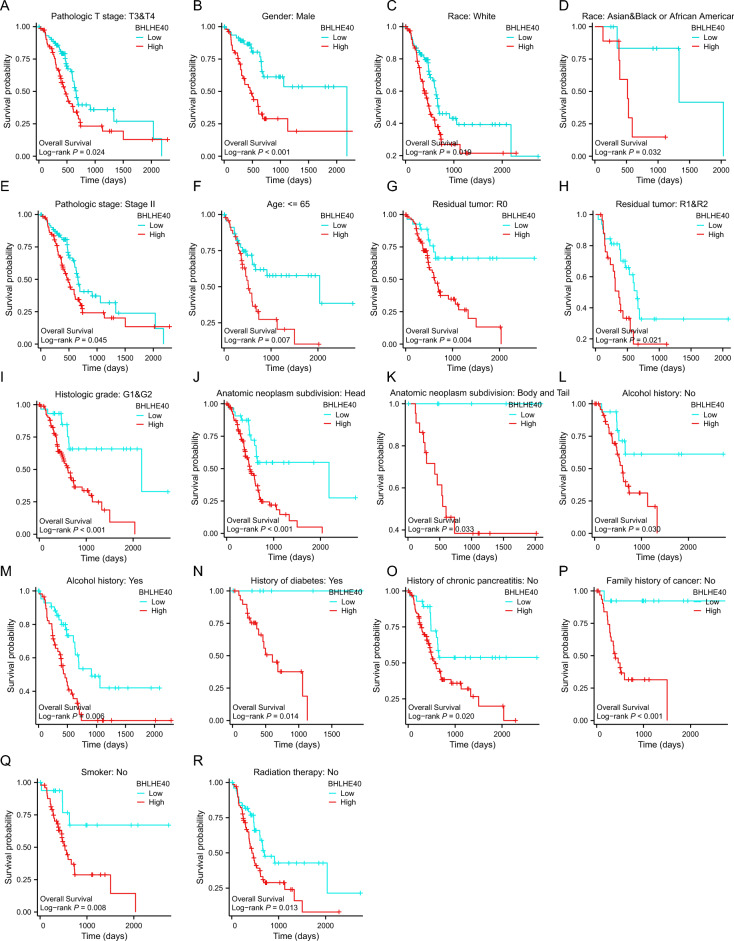
Prognostic values of BHLHE40 expression in patients with pancreatic cancer evaluated by the Kaplan–Meier method in different subgroups. Data are shown for (**A,B,F**) pathological stage; (**C**) gender; (**D,E**) race; (**G**) age; (**H,I**) residual tumor; (**J**) histologic grade; (**K,L**) anatomic neoplasm subdivision; (**M,N**) Alcohol history; (**O**) history of diabetes; (**P**) history of chronic pancreatitis; (**Q**) family history of cancer; (**R**) smoker and (**S**) radiation therapy.

To fully understand the diagnostic value of BHLHE40 for the clinical prognosis of pancreatic cancer, we revealed the association between BHLHE40, and OS based on different groups of clinical features. Our outcomes demonstrated that lower BHLHE40 expression was significantly associated with longer survival in pancreatic cancer patients in the subgroups of Pathologic T stage: T3, T3 and T4 (Fig. 4A), Gender: Male (Fig. 4B), Race: White, Asian and Black (Fig. 4C,D), Pathologic stage: Stage II (Fig. 4E), Age: ≤ 65 (Fig. 4G), Residual tumor: R0, R1 and R2 (Fig. 4F–H), Histologic grade: G1 and G2 (Fig. 4I), Anatomic neoplasm subdivision: Head, Body and Tail (Fig. 4J,K) Alcohol history (Fig. 4L,M), History of diabetes: Yes (Fig. 4N), History of chronic pancreatitis: No (Fig. 4O), Family history of cancer: No (Fig. 4QP), Smoker: No (Fig. 4Q), Radiation therapy: No (Fig. 4R). In contrast, BHLHE40 expression was not clearly related to survival of pancreatic cancer patients in subgroups such as Pathologic T stage: T1, T2 (Fig. S3A,B), Pathologic stage: Stage I (Fig. S3C), Primary therapy outcome: (PD, SD and PR and CR) (Fig. S3D,F), Gender: Female (Fig. S3E), Histologic grade: G3 and G4 (Fig. S3G), History of diabetes: No (Fig. S3H), Family history of cancer: Yes (Fig. S3I), Smoker: Yes (Fig. S3J), Radiation therapy: Yes (Fig. S3K). It implies that BHLHE40 plays a crucial role in promoting malignant progression of pancreatic cancer in many clinical features.

### Potential functions of BHLHE40-related genes

**Figure 5:**
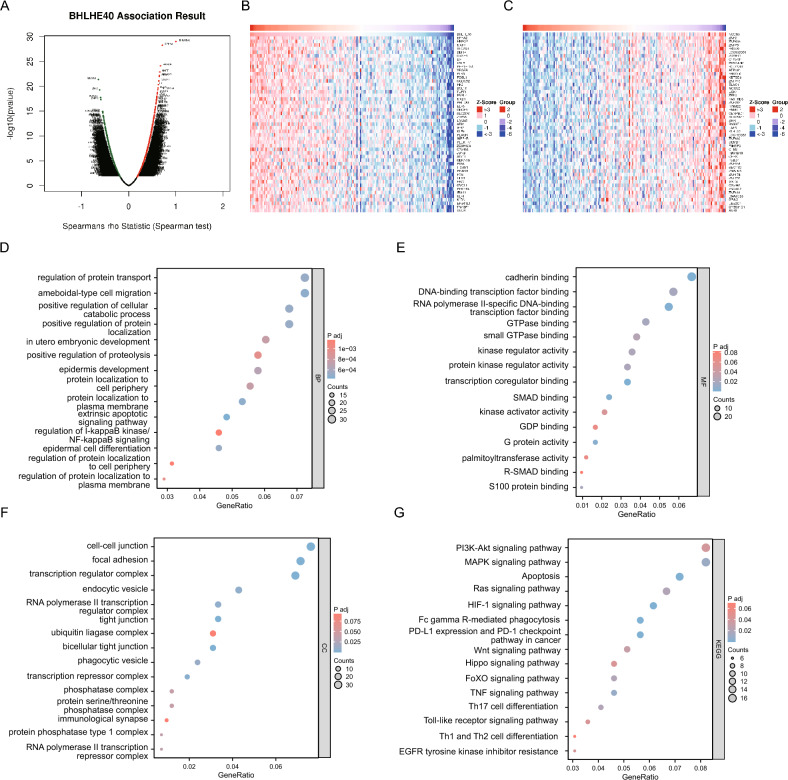
Functional enrichment analysis of BHLHE40-related genes. (**A–C**) The association result of BHLHE40-related genes. (**D–G**) GO and KEGG enrichment analysis of BHLHE40-related genes.

BHLHE40, as a pro-oncogenic transcription factor, could be involved in regulating the transcription of a variety of genes affecting tumor progression. Hence, to discover the specific functions of BHLHE40-related genes, we initially identified all genes positively and negatively associated with BHLHE40 by using LinkedOmics online software (Fig. 5A–C). Subsequently, we conducted Gene Ontology(GO) enrichment analysis of all BHLHE40-related genes and realized that these genes are mainly enriched in regulation of protein transport, ameboidal-type cell migration, positive regulation of cellular catabolic process, positive regulation of protein localization in biological processes (BP) (Fig. 5D), cadherin binding, DNA-binding transcription factor binding, RNA polymerase II-specific DNA-binding in molecular functions (MF) (Fig. 5E), and cell–cell junction, focal adhesion, transcription regulator complex, tight junction in cellular components (CC) (Fig. 5F). Regarding the pathway enrichment of BHLHE40-related genes, Kyoto encyclopedia of genes and genomes (KEGG) analysis displayed that the PI3K-AKT, Toll-like receptor, Wnt signaling pathway, PD-L1 expression and PD-1 checkpoint pathway in cancer, Th17 cell differentiation was remarkably enriched (Fig. 5G). These findings potentially implied that BHLHE40 probably plays an important function in tumor proliferation, metastasis, energy metabolism, and immune escape.

### BHLHE40 facilitates immunosuppression in the tumor microenvironment of pancreatic cancer

**Figure 6:**
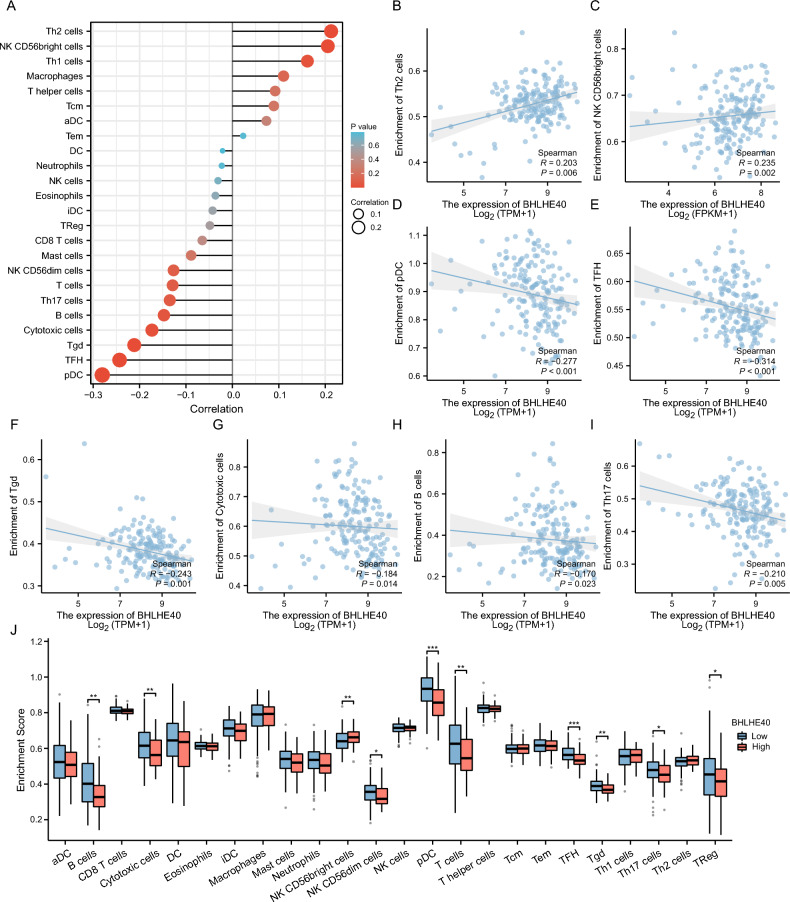
Correlation analysis of BHLHE40 expression and infiltration levels of immune cells in pancreatic cancer. Correlation between BHLHE40 expression and relative abundance of 24 types of immune cell. (**B–I**) Correlations between the relative enrichment scores of immune cells (including Th2 cells, NK CD56bright cells, pDCs, TFHs, Tgds, Cytotoxic cells, B cells and Th17 cells) and the expression of BHLHE40. (**J**) Enrichment ratio of each immune cells (B cells, Cytotoxic cells, NK CD56dim cells, pDC, T cells, TFH, Tgd and Th17 cells etc.) in samples from the BHLHE40 high expression group and low expression group.

By functional enrichment we identified that BHLHE40 potentially exerts a critical role in the TME of pancreatic cancer. For this sake, it is particularly essential to determine the connection between the expression of BHLHE40 and the degree of immune cell infiltration in pancreatic cancer. To begin with, after the TCGA database was used to examine the correlation of BHLHE40 with immune cells by combining the single-sample gene set enrichment analysis (ssGSEA) algorithm (Fig. 6A), we detected that BHLHE40 was positively correlated with Th2 cells (R = 0.203, P = 0.006) (Fig. 6B), NK CD56bright cells (R = 0.235, P = 0.002) (Fig. 6C). And Plasmacytoid Dendritic Cells (pDC) (R =  − 0.277), Follicular helper T cell (TFH) (R =  − 0.314), Tgd (R =  − 0.243), Cytotoxic cells (R =  − 0.184, P = 0.014), B cells (R =  − 0.17, P = 0.023) and Th17 cells (R =  − 0.21, P = 0.005) from pancreatic cancer patients (Fig. 6D–I) were negatively correlated to BHLHE40. In parallel, we separated pancreatic cancer patients into high and low expression groups depending on the BHLHE40 expression. After comparing the enrichment levels of each immune cell population between the two groups of patients, we observed that B cells, Cytotoxic cells, NK CD56dim cells, pDC, T cells, TFH, Tgd and Th17 cells were strongly enriched in the low expression BHLHE40 group (Fig. 6J). NK CD56bright cells were obviously abundant in the BHLHE40 high group (Fig. 6J). It implies that BHLHE40 possesses the function of suppressing immune response and increasing immune escape in pancreatic cancer.

### Diagnostic value of BHLHE40 in different types of immune cells on the prognosis of pancreatic cancer

**Figure 7:**
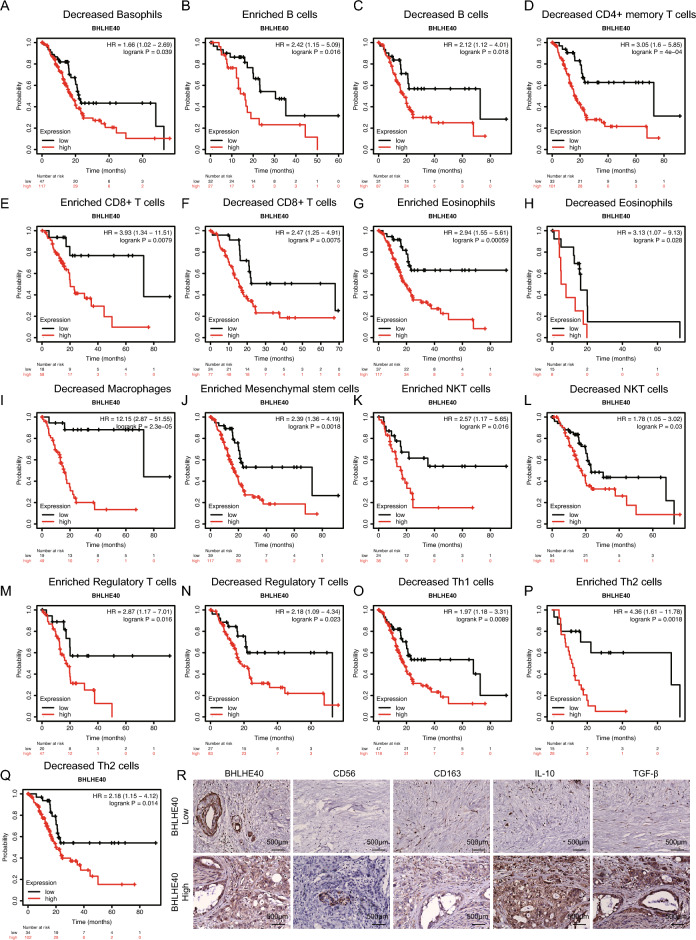
Kaplan–Meier survival curves according to the high and low expression of BHLHE40 in immune cell subgroups in pancreatic cancer. (**A–Q**) Correlations between BHLHE40 expression and overall survival in different immune cell subgroups in pancreatic cancer patients were determined by Kaplan–Meier plotter. (**R**) Immunohistochemical analysis of CD56, CD163, IL10, and TGFβ expression in tissue sections from pancreatic cancer patients with varying levels of BHLH40 expression (Scale bar, 500 μm).

We clarified the position of BHLHE40 in the TME of pancreatic cancer through the above study. Furthermore, to investigate the impact of BHLHE40 in the TME on pancreatic cancer survival, we organized an analysis of the potential linkage of BHLHE40 in various immune cell subpopulations on pancreatic cancer prognosis. It was concluded that BHLHE40 was an important factor for poor pancreatic cancer prognosis in subgroups such as Decreased Basophils (HR 1.66, P = 0.039) (Fig. 7A), Enriched B cells (HR 2.42, P = 0.016) (Fig. 7B), Decreased B cells (HR 2.12, P = 0.018) (Fig. 7C), Decreased CD4+ memory T cells (HR 3.05, P = 0.0004) (Fig. 7D), Enriched CD8+ T cells (HR 3.93, P = 0.0079) (Fig. 7E), Decreased CD8+ T cells (HR 2.47, P = 0.0075) (Fig. 7F), Enriched Eosinophils (HR 1.66, P = 0.039) (Fig. 7G), Decreased Eosinophils (HR 3.13, P = 0.028) (Fig. 7H), Decreased Macrophages (HR 12.15, P = 0.000023) (Fig. 7I), Enriched Mesenchymal stem cells (HR 2.39, P = 0.0018) (Fig. 7J), Enriched NKT cells (HR 2.57, P = 0.016) (Fig. 7K), Decreased NKT cells (HR 1.78, P = 0.03) (Fig. 7L), Enriched Regulatory T cells (HR 2.87, P = 0.016) (Fig. 7M), Decreased Regulatory T cells (HR 2.18, P = 0.023) (Fig. 7N), Decreased Th1 cells (HR 1.97, P = 0.0089) (Fig. 7O), Enriched Th2 cells (HR 4.36, P = 0.0018) (Fig. 7P), Decreased Th2 cells (HR 2.18, P = 0.014) (Fig. 7Q). And there was no clear association for pancreatic cancer prognosis in only three subgroups (Enriched CD4+ memory T cells, Enriched Macrophages and Decreased Mesenchymal stem cells) (Fig. S4A–C). In short, elevated expression of BHLHE40 in the vast majority of immune cells contributed to the occurrence of poor prognosis in pancreatic cancer patients. In addition, to elucidate the spatiotemporal information of immunosuppressive cells in the real immune microenvironment of PDAC, we confirmed that high expression of BHLHE40 in PDAC tissues was associated with a high infiltration of immunosuppressive cells by IHC assay of tumor immunosuppressive cell-specific markers (IL10, TGF-β, CD56, CD163) in combination with BHLHE40 (Fig. 7R).

### Abundant intercellular communication exists in classified immune cell subgroups with high BHLHE40 expression

**Figure 8:**
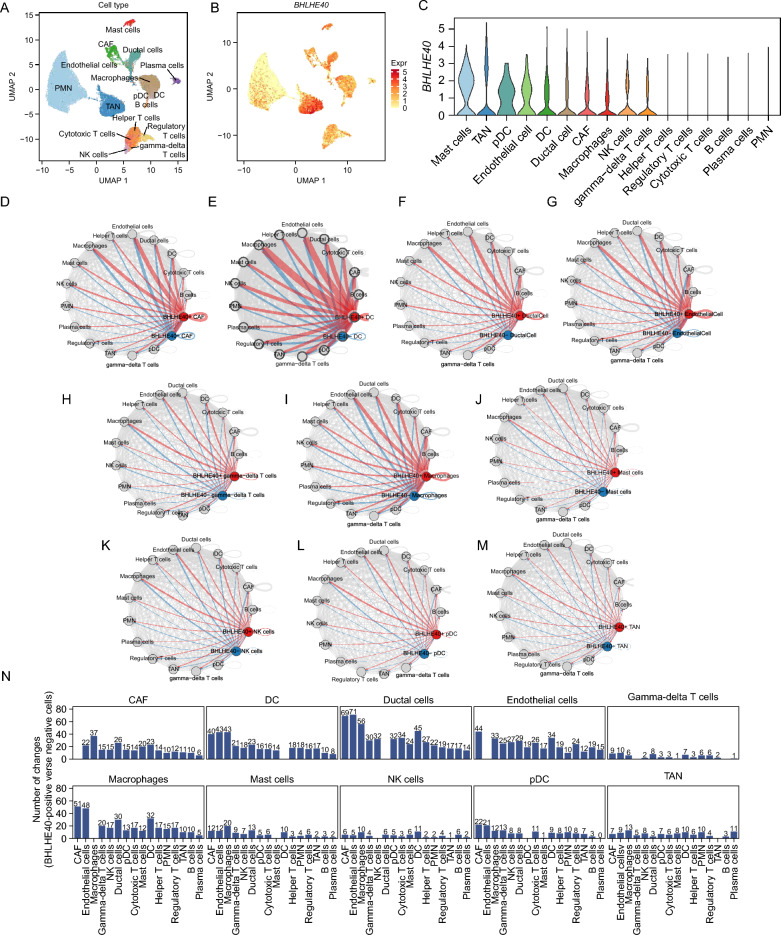
Abundant intercellular communication exists in classified immune cell subgroups with high BHLHE40 expression. (**A**) The umap plot demonstrates main cell types in PDAC. (**B**) Expression levels of BHLHE40 for all types of cells are plotted onto the umap. Color key from yellow to red indicates relative expression levels from low to high. The “expression level” was normalized by logNormalize method in Seurat. (**C**) Violin plots displaying the expression of BHLHE40 across the cell types identified in PDAC. The y axis shows the normalized read count. (**D–M**) The crosstalk between BHLHE40^+^ (Red)/BHLHE40^−^ (Blue) clusters and other clusters. (**N**) The number of changes of crosstalk between BHLHE40^+^ cells and BHLHE40^−^ cells.

To further demonstrate that BHLHE40 plays a crucial role in the tumor microenvironment of pancreatic cancer, we identified that BHLHE40 was dramatically hyper-regulated in the majority of immune cells, including mast cells, tumor-associated neutrophile (TAN), pDC, endothelial cell, dendritic cells (DC), ductal cell, cancer associated fibroblasts (CAF), macrophages, natural killer (NK) cells and gamma-delta T cells by single-cell RNA sequencing analysis (Fig. 8A–C). Subsequently, we divided each cell clusters into two subclusters with high expression of BHLHE40 and low expression of BHLHE40, and analyzed the signaling levels between these two subpopulations and other immune cells by Cellphone DB package, and discovered that the various cell clusters with high expression of BHLHE40 have more extensive intercellular communication, which implies that BHLHE40 is involved in regulating the communication between multiple immune cells in the whole tumor immune microenvironment of pancreatic cancer, and plays a role in remodeling the tumor immune microenvironment (Fig. 8D–N).

### Construction and validation of a prognostic nomogram model on BHLHE40

We performed calibration curve analysis by incorporating the parameters of BHLHE40, T-stage, N-stage, pathological stage, primary therapy outcome, residual tumor into the construction of the prognostic nomogram model for overall survival of pancreatic cancer at 1, 2, and 3 years (Fig. S5A), and found that the calibration curve was in excellent agreement with the desired model (Fig. S5B–D). This implies that our proposed nomogram has high accuracy in predicting the prognosis of pancreatic cancer patients.

### BHLHE40 facilitates the proliferation, migration, and apoptosis of pancreatic cancer cells

**Figure 9:**
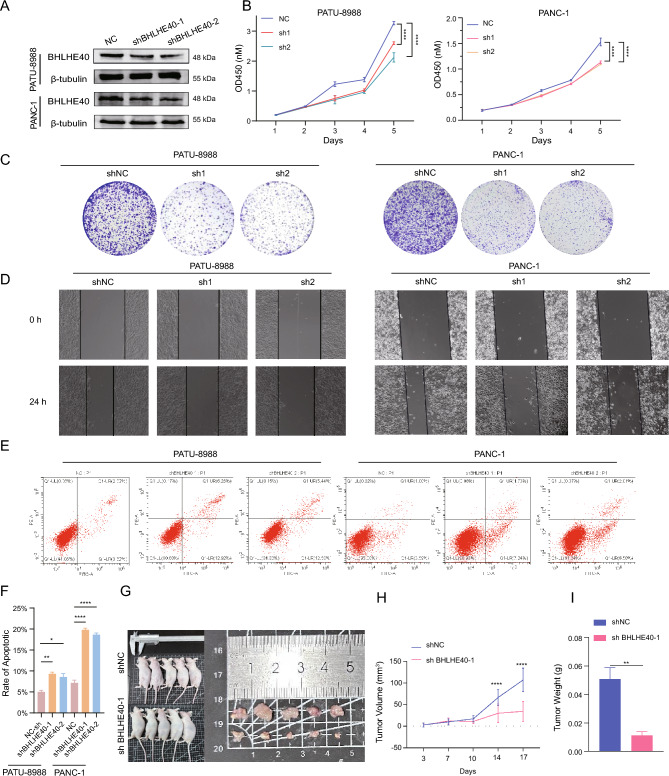
BHLHE40 facilitates the proliferation, migration and apoptosis of pancreatic cancer cells. (**A**) BHLHE40 expression in PATU-8988 cells knockdown of BHLHE40. (**B,C**) Cell Counting Kit-8 (**B**) and colony formation (**C**) were used to detect the cell viability of PATU-8988 (NC, sh1-BHLHE40 and sh2-BHLHE40) at the indicated timepoint. (**D**) Wound healing assay of PATU-8988 cells (shNC, sh1-BHLHE40, sh2-BHLHE40). Photos were taken at 0 and 24 h. (**E,F**) Flow cytometry was used to analyze the apoptosis rate in PATU-8988 cells transfected with sh1-BHLHE40, sh2-BHLHE40 and negative control. (**G**) Representative images of xenograft tumors derived from BHLHE40 knocked down and negative control PATU-8988 cells that were subcutaneously injected into BALB/c athymic nude mice (n = 5). (**H,I**) Tumor volumes and tumor weights of xenograft tumors derived from the BHLHE40 knocked down and negative control in PATU-8988 are shown. Tumor volumes were calculated as volume = length × (width)^2^/2. The data are represented as mean ± SD.

To further clarify the pro-oncogenic function of BHLHE40 on pancreatic cancer, we have firstly successfully established a BHLHE40 knockdown pancreatic cancer cell line, PATU-8988 and PANC-1(Fig. 9A). Then, we investigated that the attenuation of BHLHE40 expression could inhibit the proliferation of pancreatic cancer cells by Cell Counting Kit-8 (CCK8) assay and colony formation assay (Fig. 9B,C). Wound healing assays were utilized to prove that BHLHE40 could enhance the migration of pancreatic cancer cells (Fig. 9D). Meanwhile, we revealed that knockdown of BHLHE40 could augment the apoptosis level of pancreatic cancer cells by flow cytometry assay (Fig. 9E,F). Additionally, we observed that mice in the sh1 BHLHE40 group had lower volume and weight of tumors after subcutaneous injection of PATU-8988 cell lines (shNC and sh1 BHLHE40) into BALB/c nude mice (Fig. 9G–I). In sum, BHLHE40 exerts an influential role in boosting the malignant progression of pancreatic cancer.

### BHLHE40 is potentially regulated by the FGD5-AS1-miR-15a-5p/miR-16-5p/miR-454-3p axis

**Figure 10:**
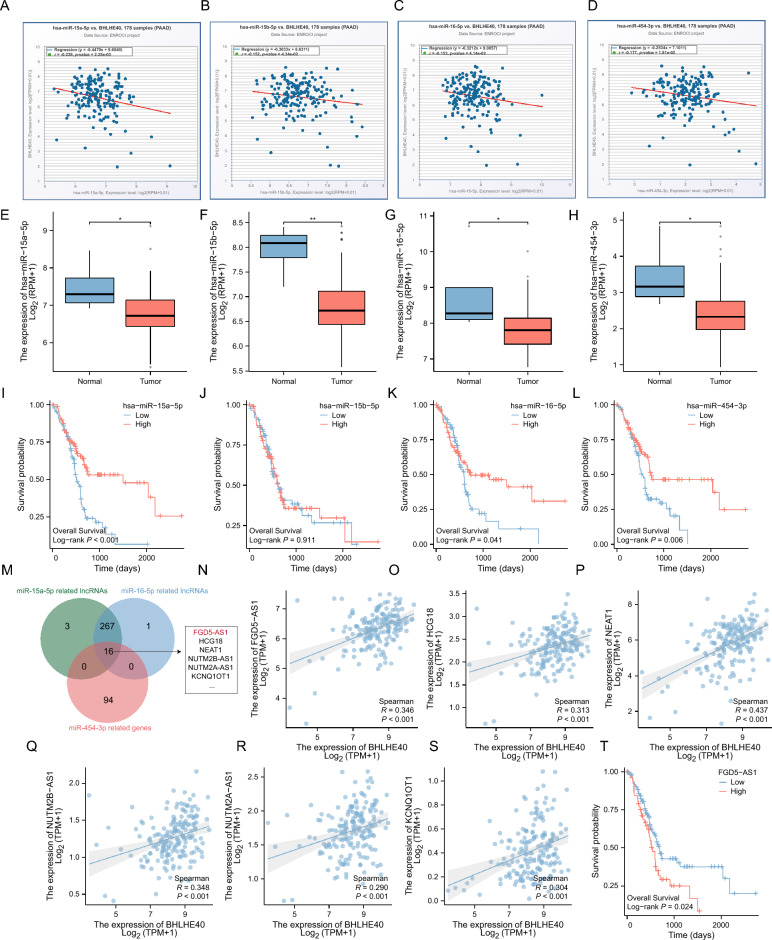
FGD5-AS1-miR-15a-5p/miR-16-5p/miR-454-3p axis regulatory network. (**A–D**) Correlations between BHLHE40 expression and miR-15a-5p, miR-15b-5p, miR-16-5p and miR-454-3p in pancreatic cancer. (**E–H**) Analysis of miR-15a-5p, miR-15b-5p, miR-16-5p and miR-454-3p expression in pancreatic cancer and adjacent normal tissues in the TCGA database. (**I–L**) The expression levels of miR-15a-5p, miR-15b-5p, miR-16-5p and miR-454-3p in pancreatic cancer patients were determined by Kaplan–Meier plotter. (**M**) The upstream lncRNAs jointly targeted by miR-15a-5p, miR-16-5p, and miR-454-3p were predicted by bioinformatics. (**N–S**) Correlations between BHLHE40 expression and FGD5-AS1, HCG18, NEAT1, NUTM2B-AS1, NUTM2A-AS1 and KCNQ1OT1 in pancreatic cancer. (**T**) Association between FGD5-AS1 expression and pancreatic cancer patients’ outcomes.

After clarifying the function of BHLHE40 in promoting malignant progression of pancreatic cancer cells, in order to explore how BHLHE40 is upregulated in pancreatic cancer, we are keenly interested in ceRNAs regulatory mechanisms among many potential mechanisms. Therefore, we will approach the ceRNAs mechanism in an attempt to uncover the critical factors regulating BHLHE40 expression. At first, we ascertained 232 candidate microRNAs that possibly target BHLHE40 by The Encyclopedia of RNA Interactomes (ENCORI) database analysis (Table S1). Subsequently, we clarified that the only statistically significant miRNAs after correlation analysis of all candidate microRNAs with BHLHE40 were miR-15a-5p (Fig. 10A), miR-15b-5p (Fig. 10B), miR-16-5p (Fig. 10C), miR-454-3p (Fig. 10D), miR-362-3p (Fig. S6A), miR-374a-5p (Fig. S6B), miR-329-3p (Fig. S6C), miR-195-5p (Fig. S6D), miR-483-3p (Fig. S6E), miR-345-3p (Fig. S6F). According to the regulatory mechanism of ceRNAs, mircoRNAs should have a negative relevant expression pattern with BHLHE40 and was lowly expressed in pancreatic cancer tumor tissues, we narrowed the range of microRNAs to miR-15a-5p (Fig. 10E), miR-15b-5p (Fig. 10F), miR-16-5p (Fig. 10G), miR-454-3p (Fig. 10H), miR-362-3p (Fig. S6G), miR-374a-5p (Fig. S6H) and miR-329-3p (Fig. S6I–L). We further narrowed the range by determining the prognostic relationship between microRNAs and pancreatic cancer patients, we finally concluded that miR-15a-5p, miR-15b-5p, miR-15b-5p, miR-16-3p and miR-454-3p (Fig. 10I–L) were the only statistically significant miRNAs. To further explore the upstream lncRNAs that jointly target miR-15a-5p, miR-16-5p, miR-454-3p, we screened 16 potential lncRNAs by bioinformatics prediction (Fig. 10M, Table S2). We sequentially performed Spearman Correlation analysis clarified that FGD5-AS1 (Fig. 10N), HCG18 (Fig. 10O), NEAT1 (Fig. 10P), NUTM2B-AS1 (Fig. 10Q), NUTM2A-AS1 (Fig. 10R), KCNQ1OT1 (Fig. 10S), MCM3AP-AS1 (Fig. S7A), AC02092.1 (Fig. S7B), and AC131009.4 (Fig. S7C) were statistically different (Fig. S7D–F). Ultimately, combining the relationship between lncRNAs and survival of pancreatic cancer patients, we found that FGD5-AS1 was most likely to act as upstream of BHLHE40 (Fig. 10T, Fig. S7J–V).

### miR-15a-5p targeted BHLHE40 to suppresses pancreatic cancer cells’ growth, migration, and apoptosis

**Figure 11:**
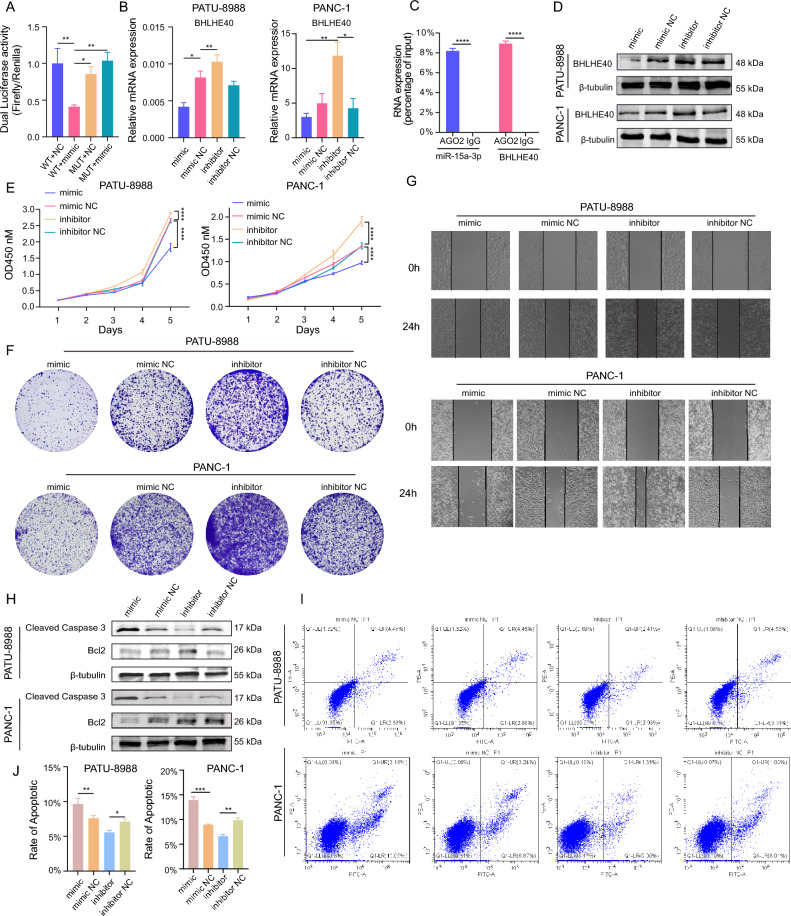
miR-15a-5p is the upstream of BHLHE40 to inhibit the growth, migration, and apoptosis of pancreatic cancer cell. (**A**) Dual-luciferase reporter assays showed the luciferase activity of PATU-8988 cells transfected with BHLHE40 overexpression plasmid and miR-15a-5p mimics or empty mimics. (**B**) qPCR was conducted to detect expression levels of BHLHE40 in PATU-8988 cells transfected with miR-15a-5p mimic or miR-15a-5p inhibitor. (**C**) AGO2 was used in the RIP assay to detect the binding level of AGO2 to miR-15a-5p or BHLHE40 in PATU-8988. (**D**) Western blot analysis showed the relative expression levels of BHLHE40 regulated by miR-15a-5p mimics and inhibitor in PATU-8988 cells. β-tubulin were used as the internal control. (**E,F**) CCK-8 and colony formation results show the proliferation rate status of mimic, inhibitor and NC in PATU-8988 cells. OD value at 450 nm. (**G**) Wound healing assay to detect the migration ability of PATU-8988 cells. Photos were taken at 0 and 24 h. (**H**) Western blot analysis showed the expression levels of Cleaved Caspase 3 and Bcl2 regulated by miR-15a-5p in PATU-8988 cells. β-tubulin were used as the internal control. (**I,J**) Flow cytometry was used to analyze the apoptosis rate in PATU-8988 cells transfected with miR-15a-5p mimics, inhibitor, and negative control.

Among the three candidate miRNAs, only miR-15a-5p was reported to have a relevant oncogenic role in pancreatic cancer, hence we concluded that miR-15-5p was the most probable upstream of BHLHE40. Further, BHLHE40 upstream was validated utilizing dual-luciferase reporter assays. The findings exhibited that PATU-8988 cells transfected with miR-15a-5p mimics (WT + miR-15a-5p mimics) had lower levels of luciferase activity than PATU-8988 cells transfected with empty mimics (WT + NC mimics group) (Fig. 11A). BHLHE40 expression was attenuated in PATU-8988 cells and PANC-1 cells after being transfected with miR-15a-5p mimics and was elevated in PATU-8988 cells and PANC-1 cells after being transfected with miR-15a-5p inhibitor (Fig. 11B). RNA immunoprecipitation (RIP) assays were used to determine whether AGO2 could serve as a scaffold to modulate the binding of miR-15a-5p and BHLHE40 in PATU-8988 cells (Fig. 11C), and the results indicated that the BHLHE40 mRNA and protein levels were downregulated in PATU-8988 cells after being transfected with miR-15a-5p mimics and was raised after miR-15a-5p inhibitor transfection in PATU-8988 cells and PANC-1 cells (Fig. 11D). To detect the miR-15a-5p function, CCK8 and colony formation assays were performed, and the results revealed decreased PATU-8988 cells and PANC-1 cells proliferation after being transfected with miR-15a-5p mimics, which was then elevated following transfection with miR-15a-5p inhibitor (Fig. 11E,F). Similarly, we observed slower wound healing in the miR-15a-5p mimics group and faster wound healing in the group of miR-15a-5p inhibitors (Fig. 11G). Further, western blotting assay and flow cytometry experiments exhibited that miR-15a-3p could increase the apoptosis of PATU-8988 cells (Fig. 11H–J).

### FGD5-AS1 acts as a ceRNA to promote BHLHE40 expression in pancreatic cancer cells

**Figure 12:**
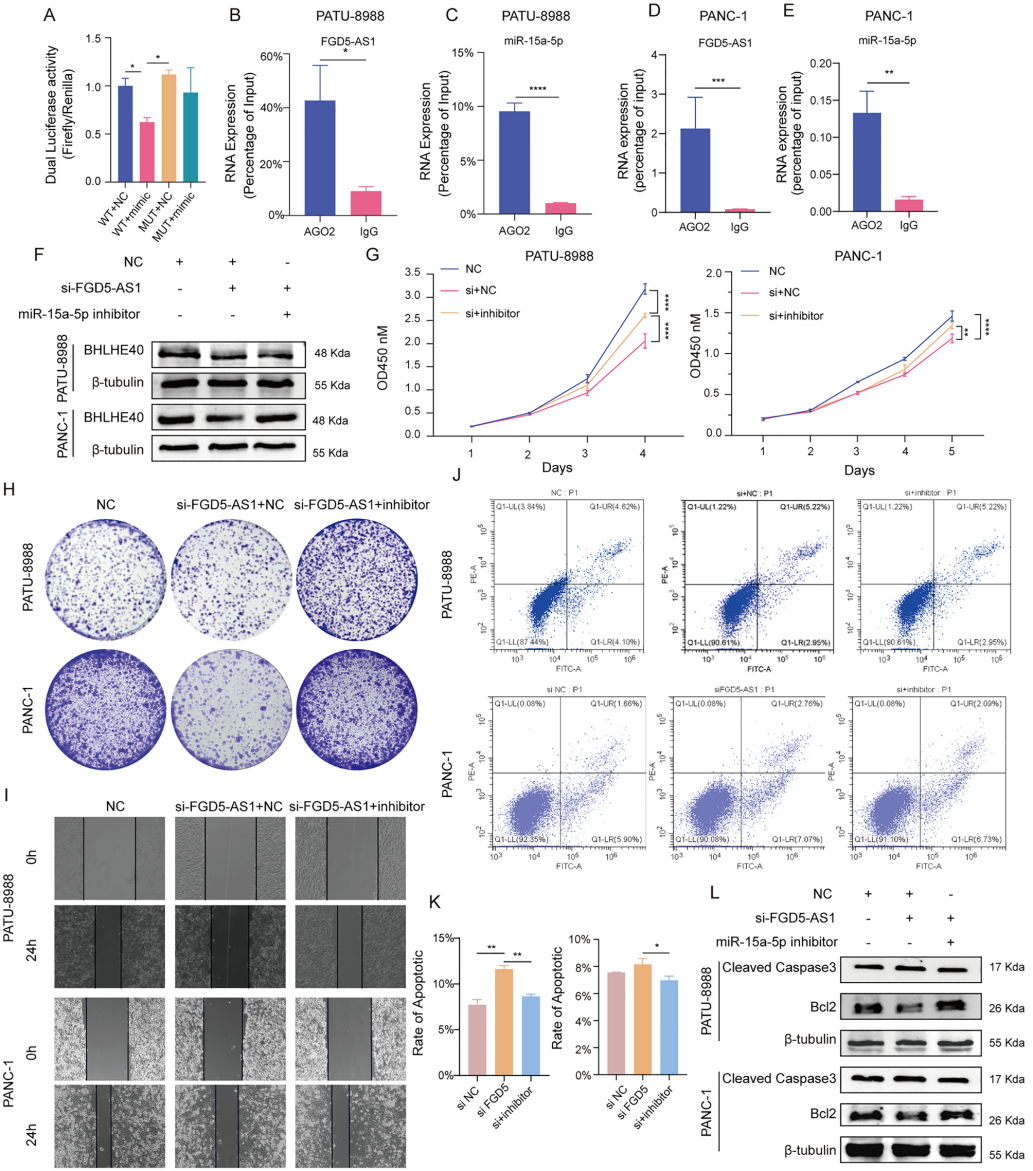
FGD5-AS1 regulates BHLHE40 expression via miR-15a-5p in pancreatic cancer cells. (**A**) Dual-luciferase reporter assays showed the luciferase activity of PATU-8988 cells transfected with FGD5-AS! overexpression plasmid and miR-15a-5p mimics or empty mimics. (**B,C**) AGO2 was used in the RIP assay to detect the binding level of AGO2 to FGD5-AS1 (**B**) and miR-15a-5p, (**C**) in PATU-8988. (**D**) The protein levels of BHLHE40 in PATU-8988 cells (NC, si-FGD5-AS1, si-FGD5-AS1 + miR-15a-5p inhibitor). (**E,F**) CCK-8 and colony formation results show the proliferation rate status of si-FGD5-AS1 + miR-15a-5p inhibitor, si-FGD5-AS1 and NC in PATU-8988 cells. OD value at 450 nm. (**G**) Wound healing assay to detect the migration ability of PATU-8988 cells (NC, si-FGD5-AS1, si-FGD5-AS1 + miR-15a-5p inhibitor). Photos were taken at 0 and 24 h. (**H**) Western blot analysis showed the expression levels of Cleaved Caspase 3 and Bcl2 regulated by FGD5-AS1/miR-15a-5p axis in PATU-8988 cells. β-tubulin were used as the internal control. (**I,J**) Flow cytometry was used to analyze the apoptosis rate in PATU-8988 cells transfected with si-FGD5-AS1, si-FGD5-AS1 + miR-15a-5p inhibitor, and negative control.

Here, dual-luciferase reporter assays were conducted to validate whether FGD5-AS1 served as a miR-15a-5p sponge in PATU-8988 (Fig. 12A). The results indicated that FGD5-AS1 and miR-15a-5p participated in AGO2-containing microribonucleoprotein complexes in PATU-8988 and PANC-1 cells (Fig. 12B–E). Western blotting experiments revealed that BHLHE40 expression was decreased after FGD5-AS1 knockdown and was rescued using si-FGD5-AS1 + miR-15a-5p inhibitor in PATU-8988 and PANC-1 cells (Fig. 12F). To investigate whether FGD5-AS1 could facilitate the growth and migration of pancreatic cancer cells via targeting miR-15a-5p, CCK8, colony formation and wound healing examinations were conducted (Fig. S8A–G, Fig. 12G–I). To further validate FGD5-AS1’s effect on the pancreatic cancer cells’ apoptosis, WB and flow cytometry experiments were performed, and the results indicated that FGD5-AS1 could attenuate the apoptosis of PATU-8988 and PANC-1 cells (Fig. S8H–O, Fig. 12J–L). Besides, in vivo the results showed that siFGD5-AS1 significantly decreased the volume and weight of subcutaneous tumors (Fig. S8P–R). In parallel, we revealed that the proliferation, migration, apoptosis promoting effects of pancreatic cancer cells by FGD5-AS1 could be reversed by miR-15a-5p, implying that FGD5-AS1 exerted its pro-tumor function by sponging miR-15a-3p (Fig. 12, Fig. S8). Specifically, to validate the activation of the FGD5-AS1/miR-15a-5p/BHLHE40 axis in pancreatic cancer in vivo, fluorescence in situ hybridization (FISH) was employed to characterize the expression of FGD5-AS1, miR-15a-5p and BHLHE40 and co-localization among these members in the tumor tissues (Fig. S9). We revealed that BHLHE40 and FGD5-AS1 were higher expressed in shNC group compared to sh1 BHLHE40 group, while miR-15a-5p expression is diametrically opposed; and there is a robust co-localization between BHLHE40 and FGD5-AS1. These findings provide evidence for the activation of the FGD5-AS1/miR-15a-5p/BHLHE40 axis during pancreatic cancer pathogenesis in vivo and its close association with tumor growth.

### Role of BHLHE40 on pancreatic cancer drug treatment sensitivity

Drug therapy tolerance in pancreatic cancer is one of the major causes of its poor prognosis^40^. For this reason, it is valuable to search for molecular markers that can predict drug sensitivity in patients. We revealed a negative correlation between BHLHE40 and the sensitivity of gemcitabine, mitomycin, paclitaxel, methotrexate, erlotinib, sunitinib, gefitinib, selumetinib, sorafenib, parp, doxorubicin, talazoparib, luminespib and trametinib after calculating the correlation between drug response to FDA-approved drugs and the expression of BHLHE40 (Fig. S10A–P). It implies that BHLHE40 attenuates the therapeutic response to these drugs and has the potential to be a predictive marker for these drugs.

## Discussion

The poor survival of cancer patients is strongly linked to the up-regulation of pro-tumor genes involved in tumor progression^41^. Due to the heterogeneity of pancreatic cancer, the use of indicators such as CA199, CA125 and cfDNA in diagnosing pancreatic cancer remains limited. Thus, it is crucial to detect new markers to predict prognosis and formulate individualized therapies. Our investigation exhibited that, in the TCGA and GTEx databases, BHLHE40 expression was elevated in multiple cancers compared to nearby healthy tissues. As a candidate transcription factor, a high level of BHLHE40 was reported to promote tumor progression in some cancers, like sarcomas, melanoma, lung, breast, colorectal and liver cancers. Nevertheless, the expression of BHLHE40 in pancreatic cancer is poorly defined, and only the molecular mechanism of BHLHE40 in tumor-associated neutrophils (TANs) has been reported in pancreatic cancer. Here, we focused on pancreatic cancer in which BHLHE40 was upregulated and connected with poor OS in different subgroups, such as race, age, alcohol history, residual tumor, pathologic stages, and gender. In addition, BHLHE40 was identified as a potential diagnostic marker for distinguishing pancreatic cancer from healthy pancreatic tissues in ROC curve analysis.

Prior investigations have informed that BHLHE40 in TANs is essential for pancreatic cancer cell proliferation, migration and the growth ability of CD8+ T cells but did not explore the underlying mechanisms and biological functions of BHLHE40 in cancers^42^. This study discovered the potential molecular pathways by which BHLHE40 influences pancreatic cancer malignant progression. GO and KEGG enrichment analysis indicated that BHLHE40 is significantly associated with altered PI3K-AKT, TNF, MAPK, PD-L1 expression and PD-1 checkpoint, regulation of protein transport, cell migration, cell–cell junction, transcription factor binding activity, RNA polymerase II transcription repressor complex. Recently, several investigations indicated that BHLHE40 could enhance the malignant development of colon tumors by stimulating the HBEGF mechanism and the hypoxia signaling axis. However, these findings demand further experimental confirmation and may diversify the relevant biological functions of BHLHE40 in pancreatic cancer.

It has been demonstrated that BHLHE40 expression is raised in TANs and correlated with poor prognosis in PDAC. Here, our results showed the possible involvement of BHLHE40 in modulating the TME of pancreatic cancer. Pancreatic cancer can lead to a high mortality rate due to its unique TME, which contains a large number of immunosuppressive cells such as TANs, TAMs and Tregs that have vital functions in facilitating tumor progression and treatment resistance. Our outcomes showed that cancer-associated immune cells, like Th2, NK CD56bright cells, positively linked with BHLHE40 expression in pancreatic cancer. PDC, TFH, Tgd, cytotoxic cells, B cells and Th17 cells were negatively associated with BHLHE40 levels in pancreatic cancer. Relevant reports showed that CD56brightNK cells are generally considered to be low cytotoxicity cytokine-producing cells. In this regard, Th2 cells facilitate tumor growth by producing pro-tumor factors such as IL-4, IL-5 and IL-13. Another study revealed that Th2 cells stimulate lung cancer metastasis by activating neutrophils through complement C3, and our findings also identified that BHLHE40 significantly correlated with Th1 and Th2 cell differentiation, Th17 cell differentiation and PD-1 checkpoint in GO and KEGG analyses. In our forthcoming work, we intend to elucidate the regulatory role of BHLHE40 in T cells, which is thought to promote malignant progression of pancreatic cancer by suppressing anti-neoplastic inflammatory responses.

To pursue an exploration of the underlying upstream mechanism of BHLHE40 upregulation in pancreatic cancer, we conducted a prediction analysis of miRNA-BHLHE40 association by coupling the correlation, expression and survival analysis of miRNA and BHLHE40, and ultimately determined that hsa-miR-15a-5p, has-miR-16-5p and has-miR-454-3p in pancreatic cancer are three upstream miRNAs targeting BHLHE40. In the same way, we found that the expression of hsa-miR-15a-5p, has-miR-16-5p and has-miR-454-3p in pancreatic cancer were negatively associated with BHLHE40. Hsa-miR-15a-5p, has-miR-16-5p and has-miR-454-3p were less expressed in pancreatic cancer tissues than in healthy tissues. KM survival analysis suggested that low expression of hsa-miR-15a-5p or has-miR-16-5p or has-miR-454-3p was obviously linked to poor prognosis of PAAD. On the basis of the ceRNA hypothesis, we assumed that the upstream miRNA ought to be negatively associated with BHLHE40 owing to its post-transcriptional negative regulatory tendency. In conjunction with the ceRNA hypothesis, expression, correlation, survival and experiment analyses yielded the conclusion that hsa-miR-15a-5p emerged as the most probable regulatory miRNA of BHLHE40 in PAAD. In a nutshell, hsa-miR-15a-5p might be negatively regulated in pancreatic cancer by targeting BHLHE40. To specify the common oncogenic upstream lncRNAs of hsa-miR-15a-5p, has-miR-16-5p and has-miR-454-3p, we initially confirmed FGD5-AS1, HCG18, NEAT1, NUTM2B-AS1, NUTM2A-AS1, KCNQ1OT1, MCM3AP-AS1, and AC02092. 1 as eight cancer-causing lncRNAs of PAAD, which were apparently positively linked with BHLHE40 by ENCORI database under ceRNA hypothesis. Subsequently, by survival, correlation and expression analysis, FGD5-AS1 was detected as the most probable upstream lncRNA of BHLHE40 in PAAD. FGD5-AS1 is a novel discovered lncRNA that has been previously shown to be overexpressed in some premalignant tumors and linked to the growth and metastasis of tumor cells in relevant research. In comparison, little studies have been done on pancreatic cancer. Consequently, we remain to further uncover its functional pathways in pancreatic cancer. In short, the FGD5-AS1/hsa-miR-15a-5p/BHLHE40 axis was determined as a potential PAAD regulatory mechanism.

In sum, this work described the clinical pertinence, tumor immune microenvironment characterization, biological functions and molecular pathways of BHLHE40 in pancreatic cancer. Silencing BHLHE40 showed favorable antitumor efficacy, underlining BHLHE40 as a hopeful predictor and potential target for pancreatic cancer therapy.

## Methods

### Dataset sources and pre-processing

Bulk-RNA sequencing data and corresponding clinical information for 33 cancer types were obtained from publicly available databases, including the TCGA dataset and the Genotype-Tissue Expression (GTEx) database (https://commonfund.nih.gov/GTEx). Furthermore, data for tumor and normal pancreatic tissues were downloaded from the TCGA-PAAD dataset (https://www.cancer.gov/ccg/research/genome-sequencing/tcga). Data analysis was performed using the R software version 3.6.3. The expression analysis and Kaplan–Meier survival curves were plotted using the “ggplot2”, “survminer” and “survival” packages in R. In addition, the gene correlation analysis was conducted using the “ggstatsplot” package in R. Correlations between quantitative parameters were evaluated using Spearman’s correlation analysis.

### Kaplan–Meier mapper analysis

The KM plotter (http://kmplot.com/analysis/) was used to investigate the prognostic role of miRNAs and lncRNAs, including the role of BHLHE40 in different cancer types.

### Construction of the nomogram

A nomogram based on the independent prognostic factors in multivariate Cox analysis was constructed using the RMS package in R (version 5.1-4) to predict the overall survival probability of pancreatic cancer patients. Furthermore, calibration curves and the concordance index (C-index) were used to assess the accuracy and performance of the nomogram.

### RNA-seq analysis and functional enrichment analysis

TCGA-PAAD patient mRNA expression data and accompanying clinical information were downloaded from UCSC Xena. The expression matrix of BHLHE40 per kilobase fragment per million (FPKM) in PDAC tissues was identified. Gene ontology (GO) and Kyoto Encyclopedia of Genes and Genomes (KEGG) analyses of BHLHE40-related genes in pancreatic cancer were performed using the Goplot (version 1.0.2), and the adjusted p < 0.01, |logFC| ≥ 0.5 were considered statistically significant. The GO functional and KEGG pathway enrichment analyses were conducted using ggplot2.

### Immune infiltration analysis

The correlation between BHLHE40 and infiltration of 24 immune cell types in the pancreatic tumor microenvironment was evaluated through single-sample gene set enrichment analysis (ssGSEA) using the gene set variation analysis (GSVA) package. Moreover, the association between the expression of BHLHE40 and infiltration of immune cell types in pancreatic cancer were analyzed using the Wilcoxon rank-sum test and Spearman’s correlation analysis.

### Single cell RNA sequencing (scRNA-seq) analysis

We collected the scRNA-seq data from OEP003254^42^. We processed fastq data using fasqc and filtered fitness sequences and eliminated low-quality reads by default settings. We then mapped the pristine data to the human reference genome (Ensemble version 91) via the STAR algorithm^43^. Seruat package was used to perform subsequent cell definition and dimensionality reduction clustering analyses.

### Analysis of cell–cell communication

Cell–cell communication was performed as described in previous studies^44^. CellPhone DB (http://www.cellphonedb.org/) used to analyze ligand-receptor signaling interactions between different subclusters of cells in the pancreatic cancer tumor microenvironment.

### miRNAs and LncRNAs interaction network

The Encyclopedia of RNA Interactomes (ENCORI) database (http://starbase.sysu.edu.cn/index.php) is a platform for showing miRNA-ncRNA and miRNA-mRNA interactions. The ENCORI database was used to predict potential upstream miRNAs and LncRNAs interacting with BHLHE40 and miR-15a-5p. In addition, the correlations between BHLHE40, miRNAs, and LncRNAs in pancreatic cancer were investigated using ENCORI.

### Cell culture and reagents

PATU-8988 and PANC-1 cells were purchased from Cell Resource Center, Shanghai Institute of Biotechnology, Chinese Academy of Sciences. PATU-8988 and PANC-1 cells were cultured in DMEM (BioChannel Biological Technology Co., Ltd.) supplemented with 10% FBS (LONSERA, Shanghai Shuangru Biology Science & Technology Co., Ltd.) and 1 × penicillin/streptomycin at 37 °C with 5% CO2, and were passaged when reaching a confluency of over 80%.

### Plasmids and stable cell lines

The lentiviruses for BHLHE40 knockdown were acquired from Shanghai Bioegene Co., Ltd. The corresponding sequences are presented in Table S3. For the knockdown of FGD5-AS1, cells were transfected with specific siRNA, negative control siRNAs was used for the control cells. PATU-8988 and PANC-1 cells were transfected with miR-15a-5p mimic and inhibitor at a concentration of 50 nM. Cells were cultured to 70% confluence in 6-well plates and were transfected by using the lipofectamine 2000 reagent (11668019, Invitrogen, USA) as instructed by the manufacturer’s protocols.

### Western blot analysis

The cells were lysed with the RIPA Lysis Buffer (Strong, without inhibitors) (K1120, APExBIO, Houston, USA). PAGE Gel Kits (P0105 LABLEAD lnc.) were used to prepare the gel for electrophoresis. The details of antibodies used are listed in Supplementary Table S4. The stripes were captured by Tanon-5200 Chemiluminescent Imaging System (Tanon, China, Shanghai).

### Cell proliferation assay

The CCK-8 (Cell count kit-8) assay (C6050, New Cell & Molecular Biotech) was conducted to determine cell proliferation. Briefly, transfected pancreatic cancer cells (2 × 10^3^) were collected after 48 h and seeded into a 96-well plate (701301, NEST Biotechnology). Subsequently, 10 µL of CCK-8 assay reagent was added at specific times and incubated for another 2 h, and the absorbance was recorded at 450 nm. To perform the colony formation assay, pancreatic cancer cells were plated in 6-well plates (1 × 10^3^ cells/well). After 2 weeks, the cells were fixed in 1% crystal violet stain solution at room temperature for 20 min, and the colonies were counted manually.

### Wound healing assays

The wound healing assays were conducted as previously reported. Cells were transfected with knockdown plasmids for 72 h and spread on 6-well plates the allowed to grow to the required confluence. Wound zones prepared with a 1000 μL pipette tip and incubated with serum-free medium. Wound zones were observed and photographed per 24 h.

### Cell apoptosis assays

Apoptosis levels were assessed by measuring Caspase-3 and Bcl2 levels, while flow cytometry was employed to detect apoptotic cell levels through annexin V/PI staining (Vazyme). Briefly, cells were cultured in 10% FBS medium after transfection with knockdown or overexpression plasmids after which apoptosis staining was performed according to the manufacturer’s instructions.

### RIP

BHLHE40 in PATU-8988 cells and PANC-1 cells growth reached 90% coverage, 1 × 10^7^ cells were collected. After washing with PBS, the cells were completely shaken and mixed in lysis solution. Anti-AGO2 antibody or IgG complex was prepared for immunoprecipitation. Then, the cell supernatants were incubated with magnetic bead-antibody complexes for 2 h at 4 °C. Now RNA was purified, and the obtained RNA was used to detect whether AGO2 could serve as a scaffold to modulate the binding of miR-15a-5p and BHLHE40 by conducting qRT-PCR.

### Dual luciferase report assay

The wild type (WT) or mutant (MUT) FGD5-AS1 3′ UTR containing binding site of miR-15a-5p were synthesized and cloned into pGL3-Basic vector. miR-15a-5p mimics or miRNA-NC and WT and MUT reporter constructs were co-transfected into cells with renilla luciferase vector. After 48 h transfection, luciferase activities were determined by a dual-luciferase reporter assay system (Vazyme).

### qPCR

BHLHE40 mRNA expression was measured using qPCR conducted in a 20 μL reaction volume consisting of the following agents: 6.8 μL cDNA, 10 μL mixture, 0.4 μL primer forward, 0.4 μL primer reverse, and 2.4 μL H_2_O. The PCR reaction was performed: one 2-min cycle at 50 °C, one 10-min cycle at 95 °C, forty cycles of 15 s at 95 °C and 1 min at 60 °C. Relative mRNA expression was calculated using the 2^(−ΔΔCt)^ method. Statistical analyses were performed using GraphPad Prism5 (GraphPad Software, CA, USA).

### In vivo assays

1 × 10^7^ stable PATU-8988(sh1-BHLHE40) and control PATU-8988 cell were subcutaneously injected into per BALB/c nude mice. After 3 weeks, the mice were killed using an intraperitoneal injection with an overdose of pentobarbital. The volume (= 1/2 × length × width × width) and weight of the tumor were calculated after three weeks. Furthermore, this study is reported in accordance with the ARRIVE guidelines (https://arriveguidelines.org).

### Statistical analysis

Data analysis was performed in R platform, GraphPad Prism 8 and Zesis. Two to six repeated experiments were performed in related figures. Data are presented as the mean ± SD, and differences between the two groups were compared using the paired two-tailed Student’s t-test, one-way ANOVA and Chi-square test.

### Ethics approval

All animal study protocols were approved (SYXK (shanghai)-2018-0027) by the Institutional Animal Care and Use Committee and carried out according to Shanghai Jiaotong University’s Animal Experimentation Regulations.

TCGA belongs to public databases. The patients involved in the database have obtained ethical approval. All methods were carried out in accordance with relevant guidelines and regulations.

### Supplementary Information

Supplementary Legends.

Supplementary Figure S1.

Supplementary Figure S2.

Supplementary Figure S3.

Supplementary Figure S4.

Supplementary Figure S5.

Supplementary Figure S6.

Supplementary Figure S7.

Supplementary Figure S8.

Supplementary Figure S9.

Supplementary Figure S10.

Supplementary Table S1.

Supplementary Table S2.

Supplementary Table S3.

Supplementary Table S4.

Supplementary Information.

## Data Availability

All relevant data are within the manuscript and its Supplementary Information. The data re-analyzed during the current study are available in the TCGA database with the links of (https://www.cancer.gov/ccg/research/genome-sequencing/tcga) and the Genotype-Tissue Expression (GTEx) database (https://commonfund.nih.gov/GTEx). Publicly available datasets were analyzed in this study. This data can be found here: GSE28735 (https://www.ncbi.nlm.nih.gov/geo/query/acc.cgi?acc=GSE28735) and GSA: CRA001160 (https://ngdc.cncb.ac.cn/gsa/browse/CRA001160).
