# Supplementary material for: BHLHE40, a potential immune therapy target, regulated by FGD5-AS1/miR-15a-5p in pancreatic cancer
Source: Sci Rep. 2023 Sep 29;13:16400. doi: 10.1038/s41598-023-43577-x (PMC10541890; doi:10.1038/s41598-023-43577-x)
Supplement: Supplementary file 1 — Supplementary Legends. [file 41598_2023_43577_MOESM1_ESM.docx]

**Supplementary Table and Figure legends**

**Table S1** The potential upstream miRNAs of BHLHE40

**Table S2** The potential upstream lncRNAs of miR-15a-5p, miR-16-5p and miR-454-3p

**Table S3** Sequences of lentivirus targeting related genes

**Table S4** Antibodies used for assays

**Figure S1 Prognostic analysis of BHLHE40 mRNA expression levels in pan-cancer.**

(A-O) Prognostic analysis of BHLHE40 mRNA expression levels in ACC, BLAC, COAD, HNSC, KIRC, LIHC, LUAD, LUSC, PRAD, READ, STAD, SKCM, THCA, THYM and UCS.

**Figure S2 KM and expression analysis of BHLHE40 mRNA expression levels in pancreatic cancer.**

(A-B) Kaplan Meier survival curve presenting the disease specific survival (DSS) and Progress Free Interval (PFI) of PDAC patients by the TCGA-PDAC dataset.

(C-I) The expression of BHLHE40 in subgroups of PDAC patients including Gender (C), Age (D), Alcohol history (E), History of chronic pancreatitis (F), Family history of cancer (G), History of diabetes (H), and Pathologic N stage (I).

**Figure S3 Prognostic prediction of BHLHE40 expression in patients with pancreatic cancer evaluated by Kaplan Meier analysis in different subgroups.**

Data are shown for (A-B) Pathological T stage; (C) Pathological stage; (D, E) Primary therapy outcome; (F) Gender; (G) Histologic grade; Race; (H) History of diabetes; (I) Family history of cancer; (J) Smoker; (K) Radiation therapy.

**Figure S4 Kaplan Meier curves in immune cell subgroups in pancreatic cancer.**

(A-C) Relationship between BHLHE40 expression and overall survival in subgroups including enriched CD4+ memory T cells (A), enriched macrophages (B), decreased mesenchymal stem cells (C) in pancreatic cancer patients were determined by Kaplan Meier plotter.

**Figure S5 A nomogram and calibration curves for prediction of one-, two-, three-year overall survival rates of patients with pancreatic cancer.**

(A) A nomogram for prediction of one-, two-, three-year overall survival rates of patients with pancreatic cancer.

(B–D) Calibration curves of the nomogram prediction of one-, three-, one-, two-, three-year overall survival rates of patients with pancreatic cancer.

**Figure S6 The correlation between other candidate miRNAs and BHLHE40, and the expression of candidate miRNAs in PAAD tissues from TCGA datasets.**

(A-F) The spearman correlation between miR-362-3p (A) or miR-374a-5p (B) or miR-329-3p (C) or miR-195-5p (D) or miR-483-3p (E) or miR-345-3p (F) and BHLHE40 in PAAD.

(G-L) The expression of miR-362-3p (G) or miR-374a-5p (H) or miR-329-3p (I) or miR-195-5p (J) or miR-483-3p (K) or miR-345-3p (L) in tumor tissues and normal tissues from PDAC patients. **P* < 0.05; ***P* < 0.01; ****P* < 0.001; *****P* < 0.0001.

**Figure S7 The correlation between other candidate LncRNAs and BHLHE40, and the prognostic value of candidate LncRNAs in PDAC.**

(A-F) The spearman correlation between MCM3AP-AS1 (A) or AC02092.1 (B) or AC131009.4 (C) or TMEM132D−AS1 (D) or Z95331.1(E) or AC016717.2 (F) or LINC00852 (G) or XIST (H) or AL031595.3 (I) and BHLHE40 in PAAD.

(J-V) KM curves of candidate LncRNAs in PDAC patients.

**Figure S8** **FGD5-AS1 facilitates the proliferation, migration and apoptosis of pancreatic cancer cells**

(A-D) BHLHE40 and FGD5-AS1 expression in PATU-8988 cells and PANC-1 cells silenced of FGD5-AS1.

(E-G) Cell Counting Kit-8 (C) and colony formation (D) were used to detect the cell viability of PATU-8988 and PANC-1 cells (NC, si-FGD5-AS1) at the indicated timepoint.

(H, I) Wound healing assay of PATU-8988 cells and PANC-1 cells (NC, si-FGD5-AS1). Photos were taken at 0 and 24 hours.

(J, K) Western blot analysis showed the expression levels of Cleaved Caspase 3 and Bcl2 regulated by FGD5-AS1 in PATU-8988 cells. β-tubulin were used as the internal control.

(L-O) Flow cytometry was used to analyze the apoptosis rate in PATU-8988 cells and PANC-1 cells transfected with si-FGD5-AS1 and negative control.

(P) Representative images of subcutaneous tumors derived from FGD5-AS1 knocked down and negative control PATU-8988 cells that were subcutaneously injected into BALB/c athymic nude mice. (n=5)

(Q, R) Tumor volumes and tumor weights of xenograft tumors derived from the FGD5-AS1 knocked down and negative control in PATU-8988 are shown. Tumor volumes were calculated as volume = length × (width)^2^/2. The data are represented as mean ± SD.

**Figure S9 The association between BHLHE40 and drug sensitivity in PC**

(A, B) FISH detection of FGD5-AS1(A) or miR-15a-5p(B) and BHLHE40 in Subcutaneous tumor formation in mice was achieved by injection of shBHLHE40 lentivirus or NC PATU-8988 cells. (Postive in situ hybridization signals are visualized in green, BHLHE40 are visualized in red, blue depicts DAPI nuclear stain. Scale bar, 50 μm.)

**Figure S10 The association between BHLHE40 and drug sensitivity in PC**

(A-P) Scatter plots show the correlation of BHLHE40 expression with drug sensitivity in PC.
