# Supplementary figures and images for: BHLHE40, a potential immune therapy target, regulated by FGD5-AS1/miR-15a-5p in pancreatic cancer
Source: Sci Rep. 2023 Sep 29;13:16400. doi: 10.1038/s41598-023-43577-x (PMC10541890; doi:10.1038/s41598-023-43577-x)

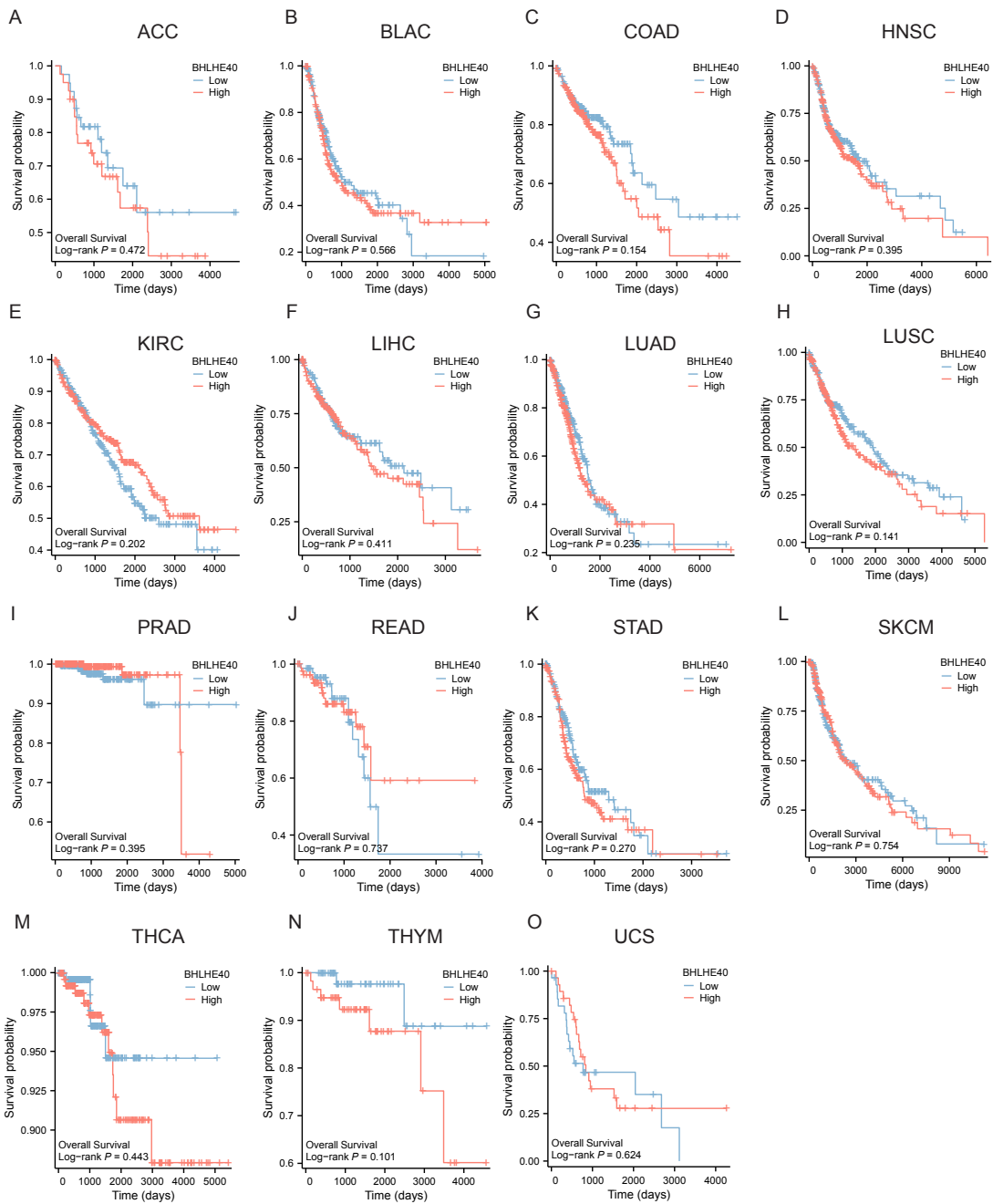

Supplement: Supplementary file 2 — Supplementary Figure S1. [file 41598_2023_43577_MOESM2_ESM.pdf]

A

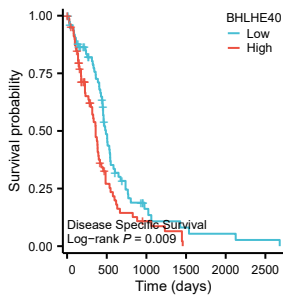

B

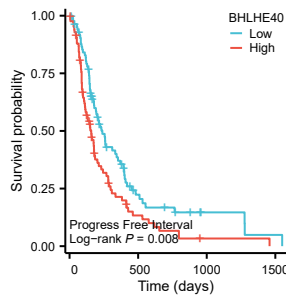

C

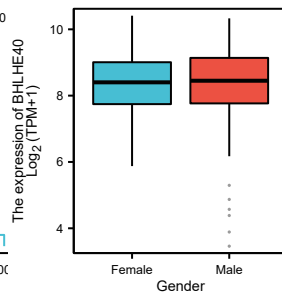

D

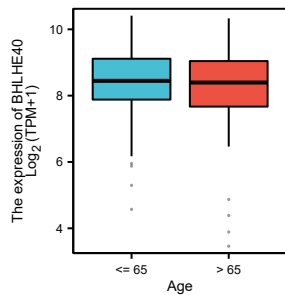

E

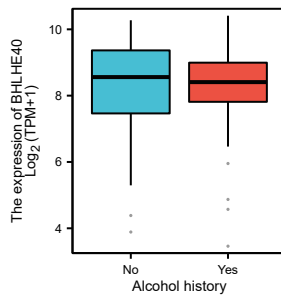

F

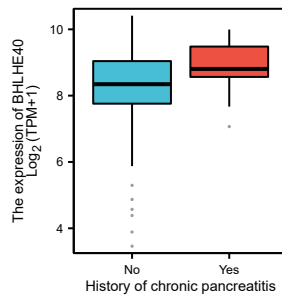

G

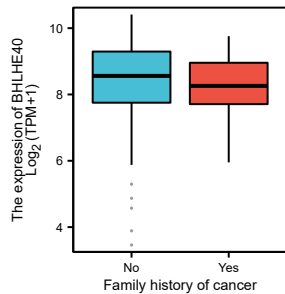

H

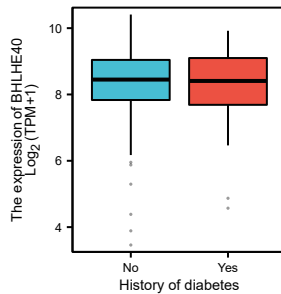

I

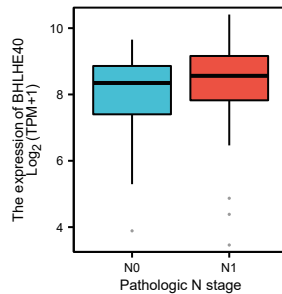

Supplement: Supplementary file 3 — Supplementary Figure S2. [file 41598_2023_43577_MOESM3_ESM.pdf]

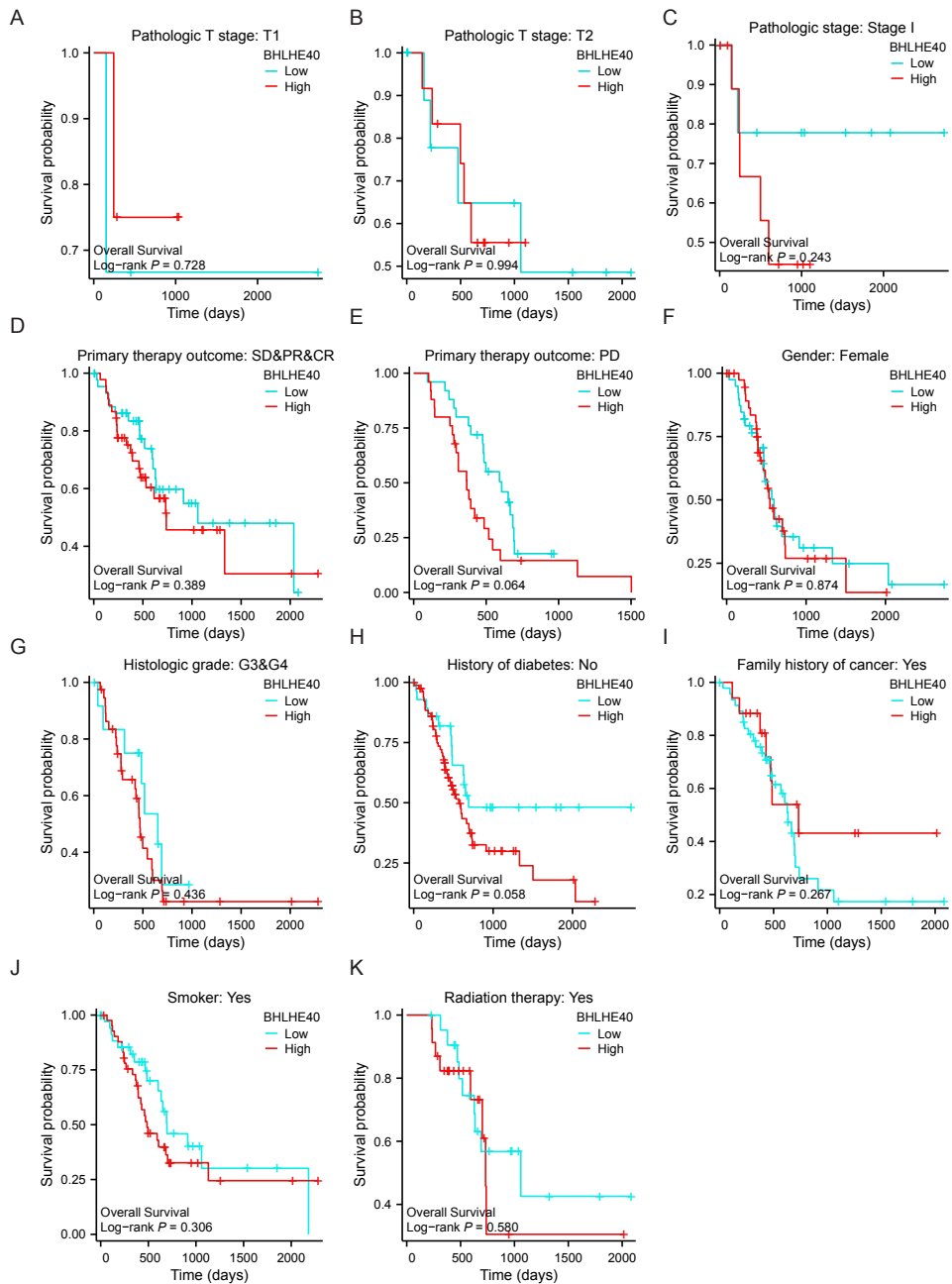

Supplement: Supplementary file 4 — Supplementary Figure S3. [file 41598_2023_43577_MOESM4_ESM.pdf]

A

Enriched CD4+ memory T cells

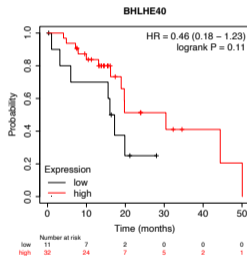

B

Enriched Macrophages

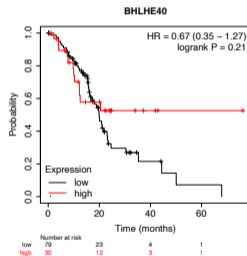

C

Decreased Mesenchymal stem cells

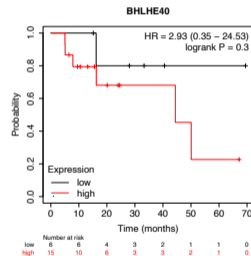

Supplement: Supplementary file 5 — Supplementary Figure S4. [file 41598_2023_43577_MOESM5_ESM.pdf]

A

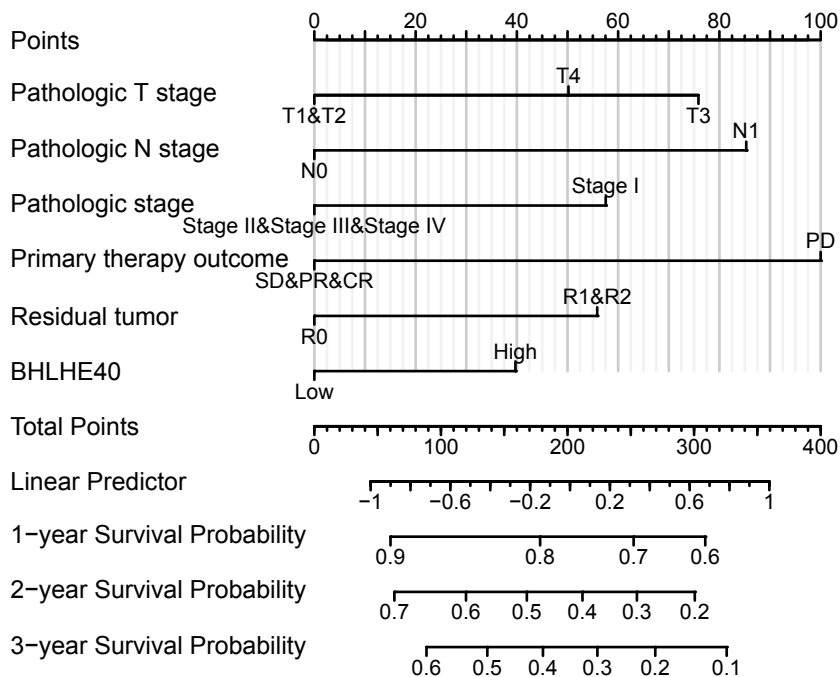

B

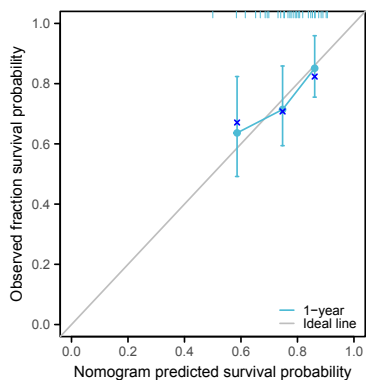

C

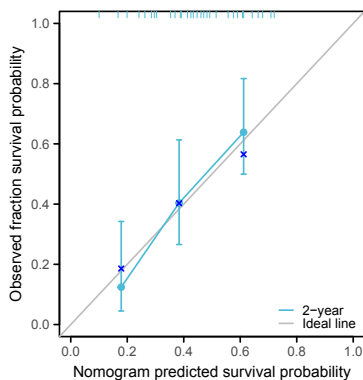

D

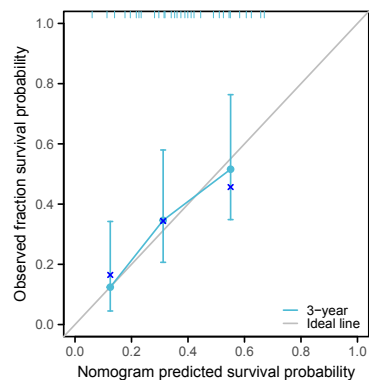

Supplement: Supplementary file 6 — Supplementary Figure S5. [file 41598_2023_43577_MOESM6_ESM.pdf]

A

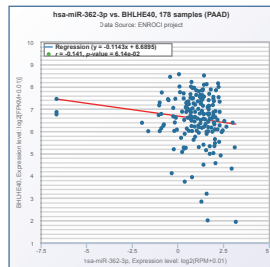

B

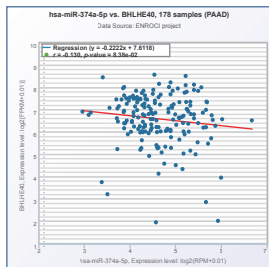

C

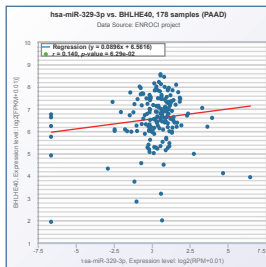

D

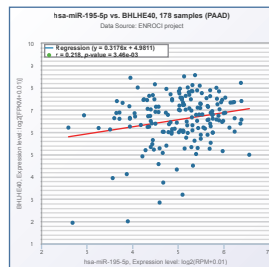

E

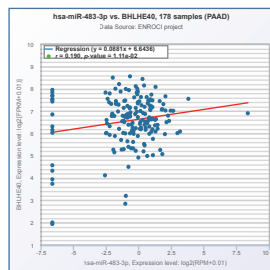

F

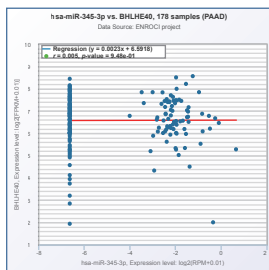

G

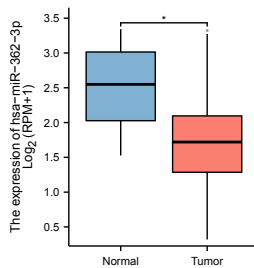

H

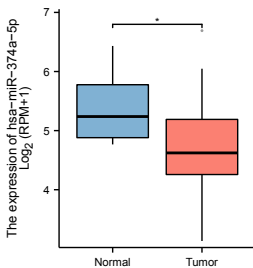

I

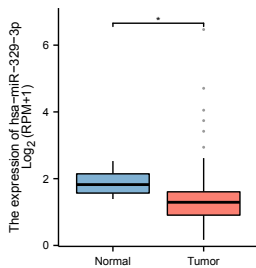

J

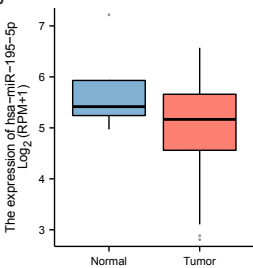

K

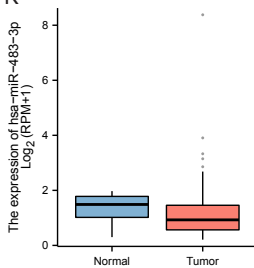

L

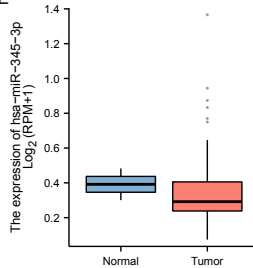

Supplement: Supplementary file 7 — Supplementary Figure S6. [file 41598_2023_43577_MOESM7_ESM.pdf]

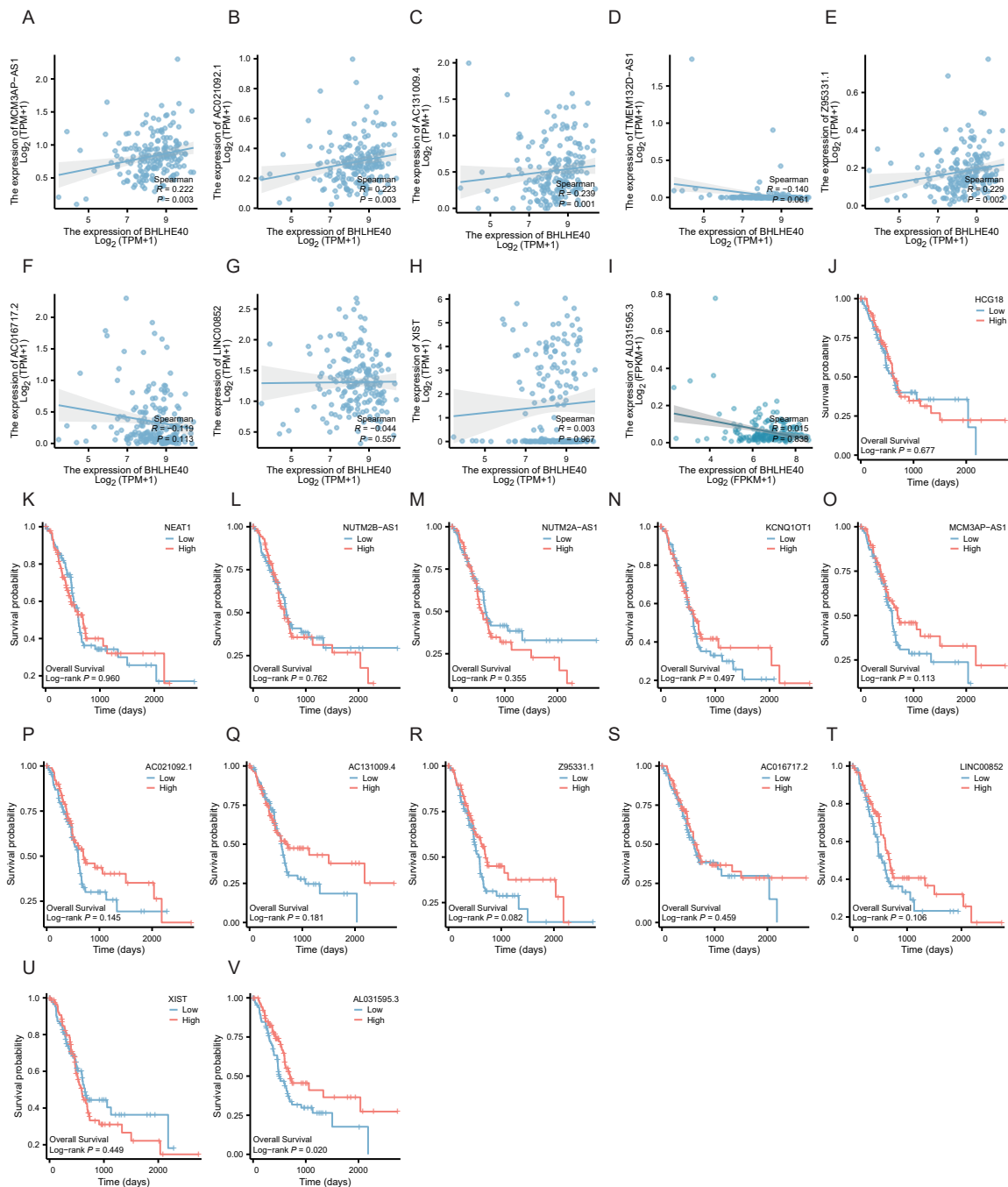

Supplement: Supplementary file 8 — Supplementary Figure S7. [file 41598_2023_43577_MOESM8_ESM.pdf]

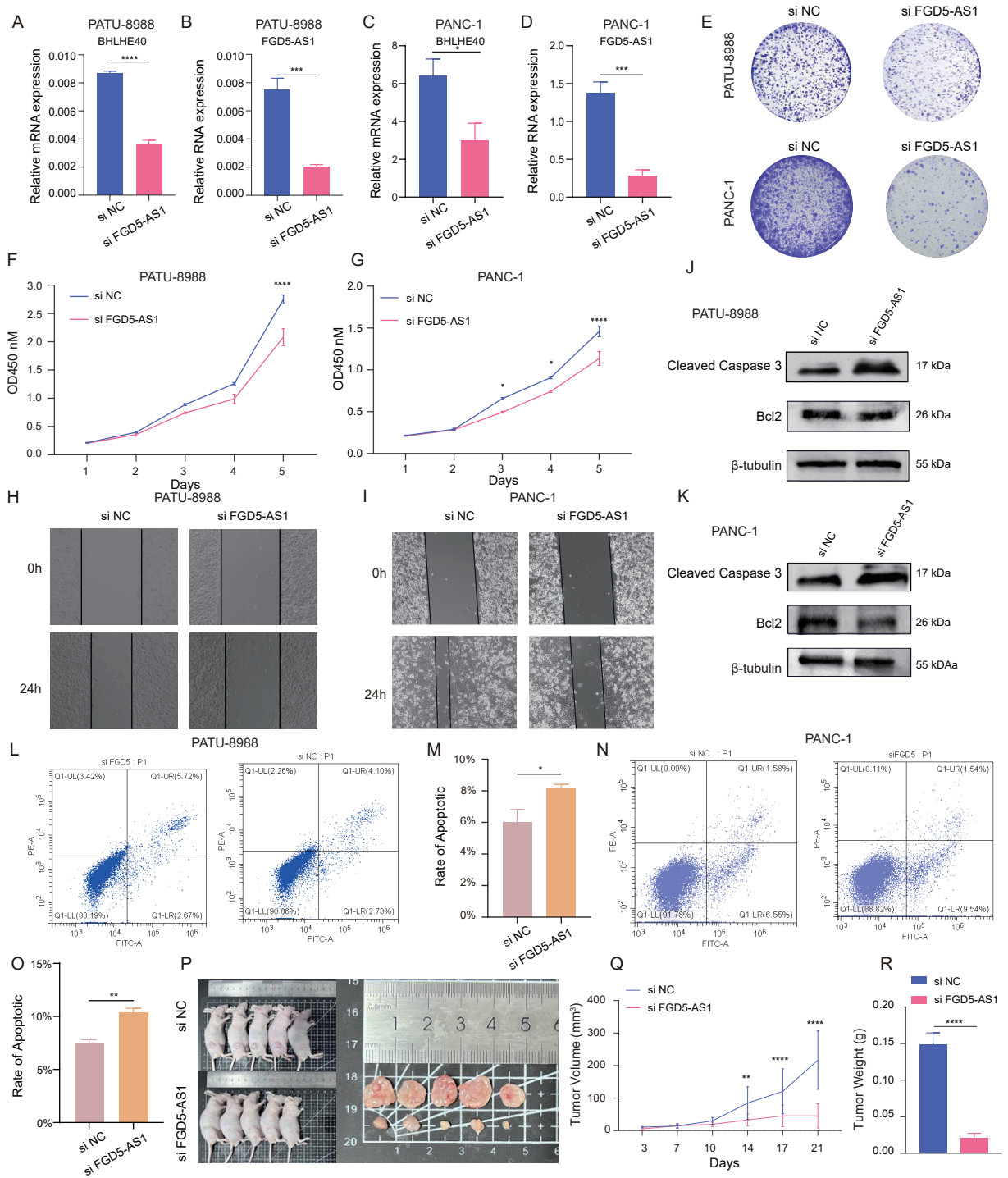

Supplement: Supplementary file 9 — Supplementary Figure S8. [file 41598_2023_43577_MOESM9_ESM.pdf]

A

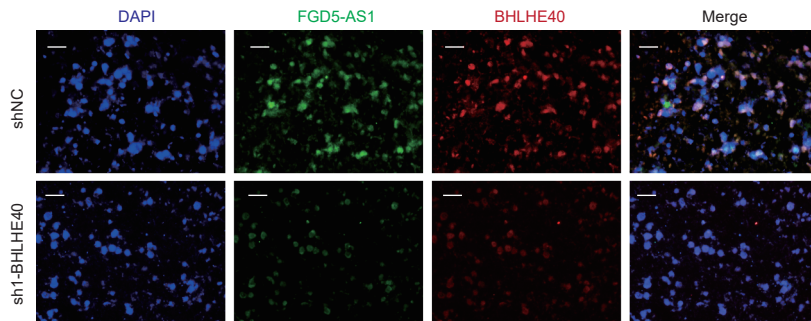

B

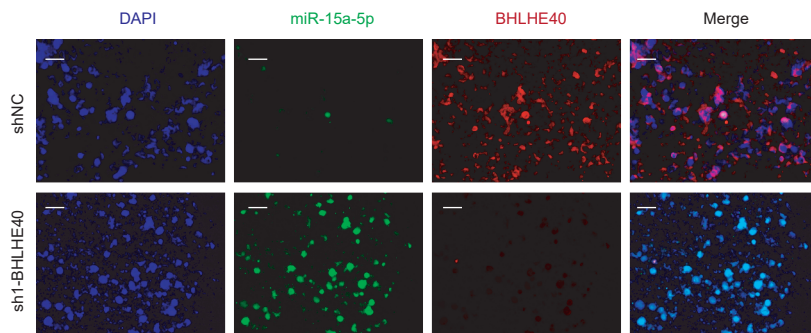

Supplement: Supplementary file 10 — Supplementary Figure S9. [file 41598_2023_43577_MOESM10_ESM.pdf]

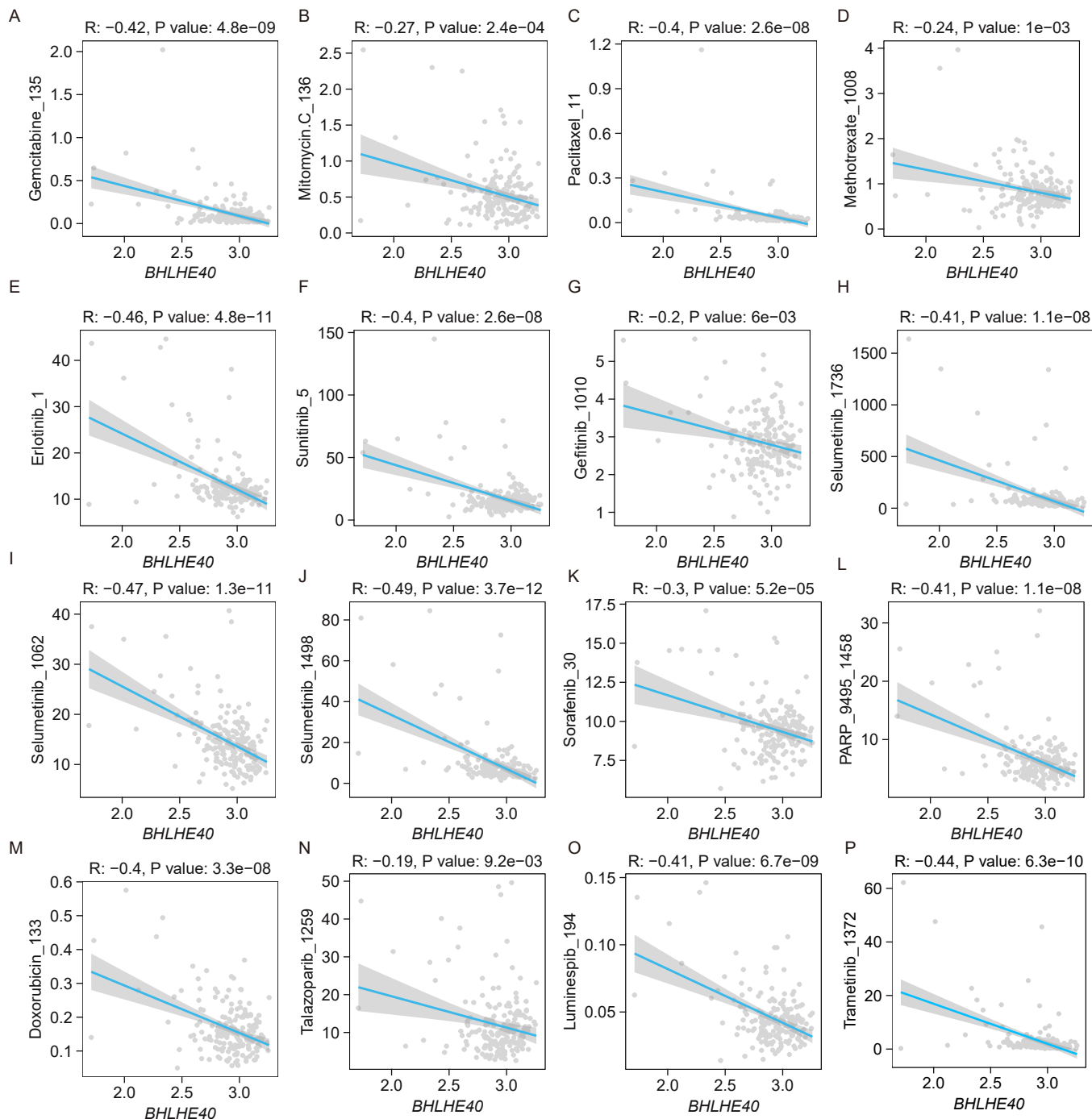

Supplement: Supplementary file 11 — Supplementary Figure S10. [file 41598_2023_43577_MOESM11_ESM.pdf]
