# Supplementary material for: BHLHE40, a potential immune therapy target, regulated by FGD5-AS1/miR-15a-5p in pancreatic cancer
Source: Sci Rep. 2023 Sep 29;13:16400. doi: 10.1038/s41598-023-43577-x (PMC10541890; doi:10.1038/s41598-023-43577-x)
Supplement: Supplementary file 14 — Supplementary Table S3. [file 41598_2023_43577_MOESM14_ESM.docx]

| **Table S3 Sequences of lentivirus targeting related genes** | | | | | |
| --- | --- | --- | --- | --- | --- |
| **ID** | **5’** | **stem** | **loop** | **stem** | **3’** |
| BHLHE40-RNAi(sh1)-a | Ccgg | GCCCACATGTACCAAGTGTAC | CTCGAG | GTACACTTGGTACATGTGGGC | TTTTTg |
| BHLHE40-RNAi(sh1)-b | aattcaaaaa | GCCCACATGTACCAAGTGTAC | CTCGAG | GTACACTTGGTACATGTGGGC |  |
| BHLHE40-RNAi(sh2)-a | Ccgg | GCAGTGGTTCTTGAACTTACC | CTCGAG | GGTAAGTTCAAGAACCACTGC | TTTTTg |
| BHLHE40-RNAi(sh2)-b | Ccgg | GCAGTGGTTCTTGAACTTACC |  | GGTAAGTTCAAGAACCACTGC |  |
