# Supplementary material for: BHLHE40, a potential immune therapy target, regulated by FGD5-AS1/miR-15a-5p in pancreatic cancer
Source: Sci Rep. 2023 Sep 29;13:16400. doi: 10.1038/s41598-023-43577-x (PMC10541890; doi:10.1038/s41598-023-43577-x)
Supplement: Supplementary file 15 — Supplementary Table S4. [file 41598_2023_43577_MOESM15_ESM.docx]

| **Table S4 Antibodies used for assays** | | | |
| --- | --- | --- | --- |
| **Antibody** | **Company** | **Cat. No.** | **Species** |
| GAPDH | Bioss | bs-0755R | Mouse |
| BHLHE40 | Abclonal | A6534 | Rabbit |
| cleaved caspase 3 | Abclonal | A11040 | Rabbit |
| Bcl2 | Abways | F084508 | Rabbit |
| Beta-tubulin | Abways | F084106 | Rabbit |
