# Supplementary material for: BHLHE40, a potential immune therapy target, regulated by FGD5-AS1/miR-15a-5p in pancreatic cancer
Source: Sci Rep. 2023 Sep 29;13:16400. doi: 10.1038/s41598-023-43577-x (PMC10541890; doi:10.1038/s41598-023-43577-x)
Supplement: Supplementary file 16 — Supplementary Information. [file 41598_2023_43577_MOESM16_ESM.pdf]

Figure S8

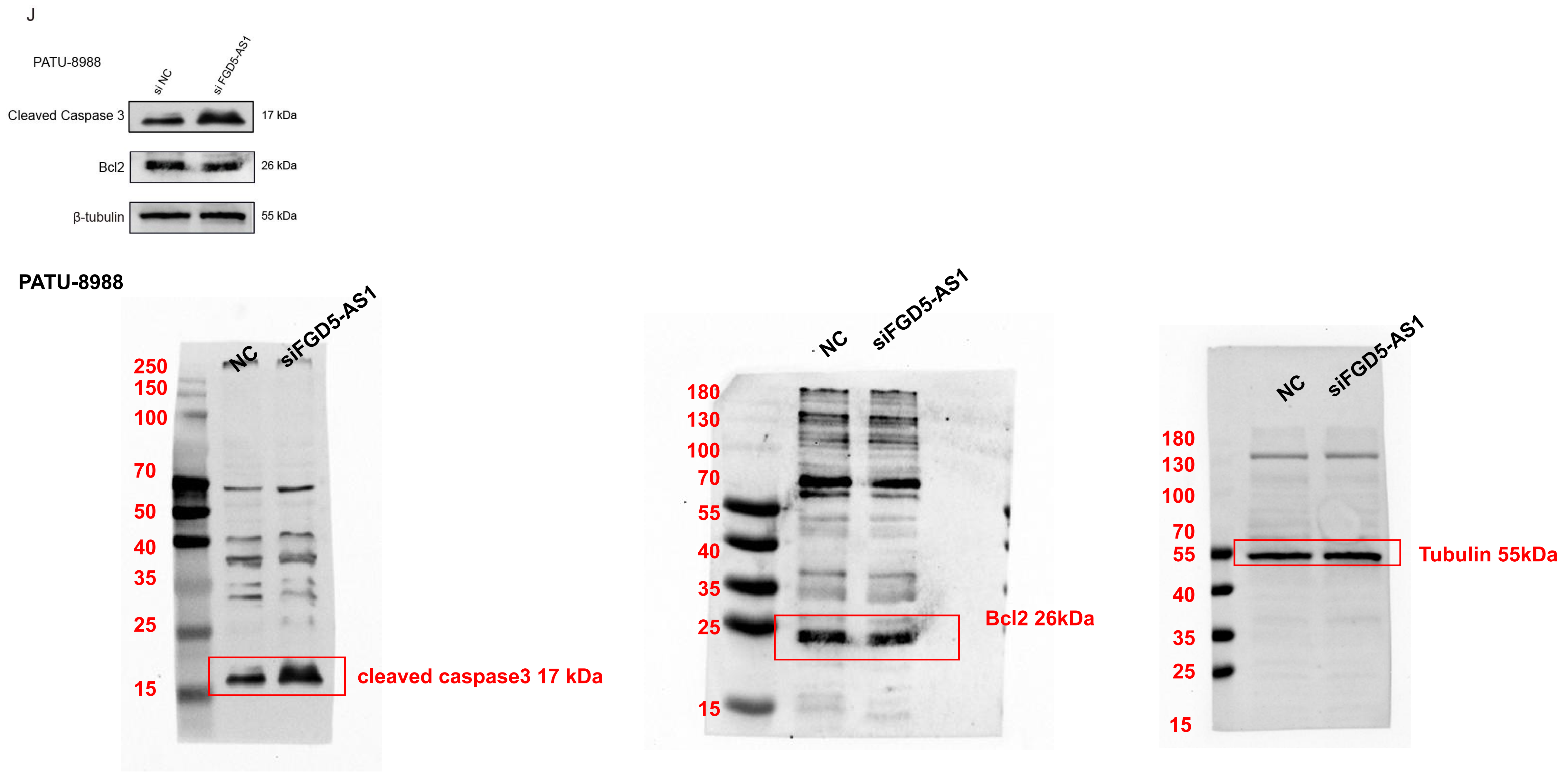
J
PATU-8988
si NC
si FGD5-AS1
Cleaved Caspase 3
17 kDa
Bcl2
26 kDa
β-tubulin
55 kDa
PATU-8988
NC
siFGD5-AS1
250
150
100
70
50
40
35
25
15
cleaved caspase3 17 kDa
NC
siFGD5-AS1
180
130
100
70
55
40
35
25
15
Bcl2 26kDa
NC
siFGD5-AS1
180
130
100
70
55
40
35
25
15
Tubulin 55kDa

Figure S8

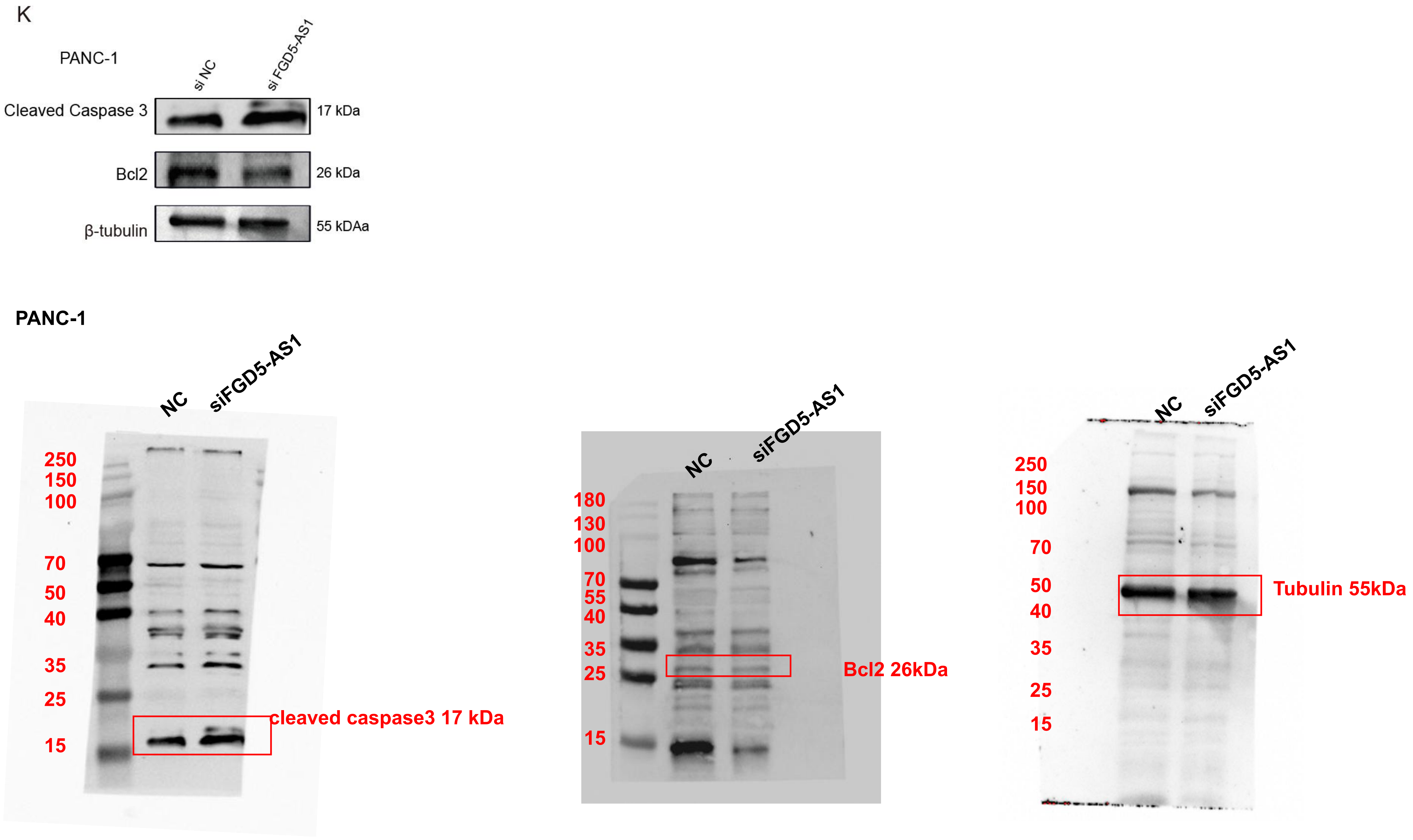
K
PANC-1
si NC
si FGD5-AS1
Cleaved Caspase 3
17 kDa
Bcl2
26 kDa
β-tubulin
55 kDAa
PANC-1
NC
siFGD5-AS1
250
150
100
70
50
40
35
25
15
cleaved caspase3 17 kDa
NC
siFGD5-AS1
180
130
100
70
55
40
35
25
15
Bcl2 26kDa
NC
siFGD5-AS1
250
150
100
70
50
40
35
25
15
Tubulin 55kDa

Figure 9

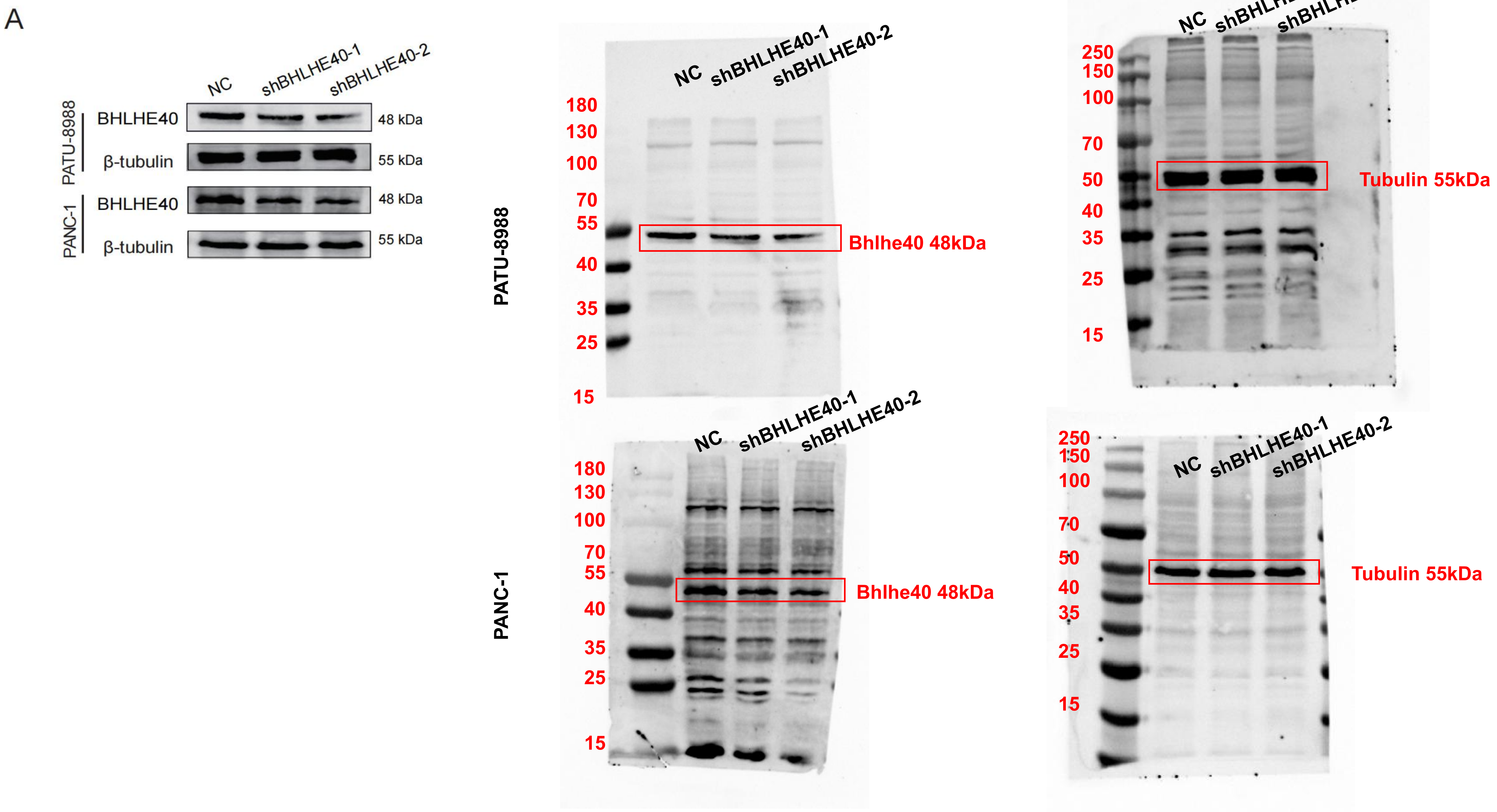
A
NC
shBHLHE40-1
shBHLHE40-2
PATU-8988
BHLHE40
48 kDa
β-tubulin
55 kDa
PANC-1
BHLHE40
48 kDa
β-tubulin
55 kDa
PATU-8988
NC
shBHLHE40-1
shBHLHE40-2
180
130
100
70
55
40
35
25
15
Bhlhe40 48kDa
NC
shBHLHE40-1
shBHLHE40-2
250
150
100
70
50
40
35
25
15
Tubulin 55kDa
PANC-1
NC
shBHLHE40-1
shBHLHE40-2
180
130
100
70
55
40
35
25
15
Bhlhe40 48kDa
250
150
100
70
50
40
35
25
15
NC
shBHLHE40-1
shBHLHE40-2
Tubulin 55kDa

Figure 11

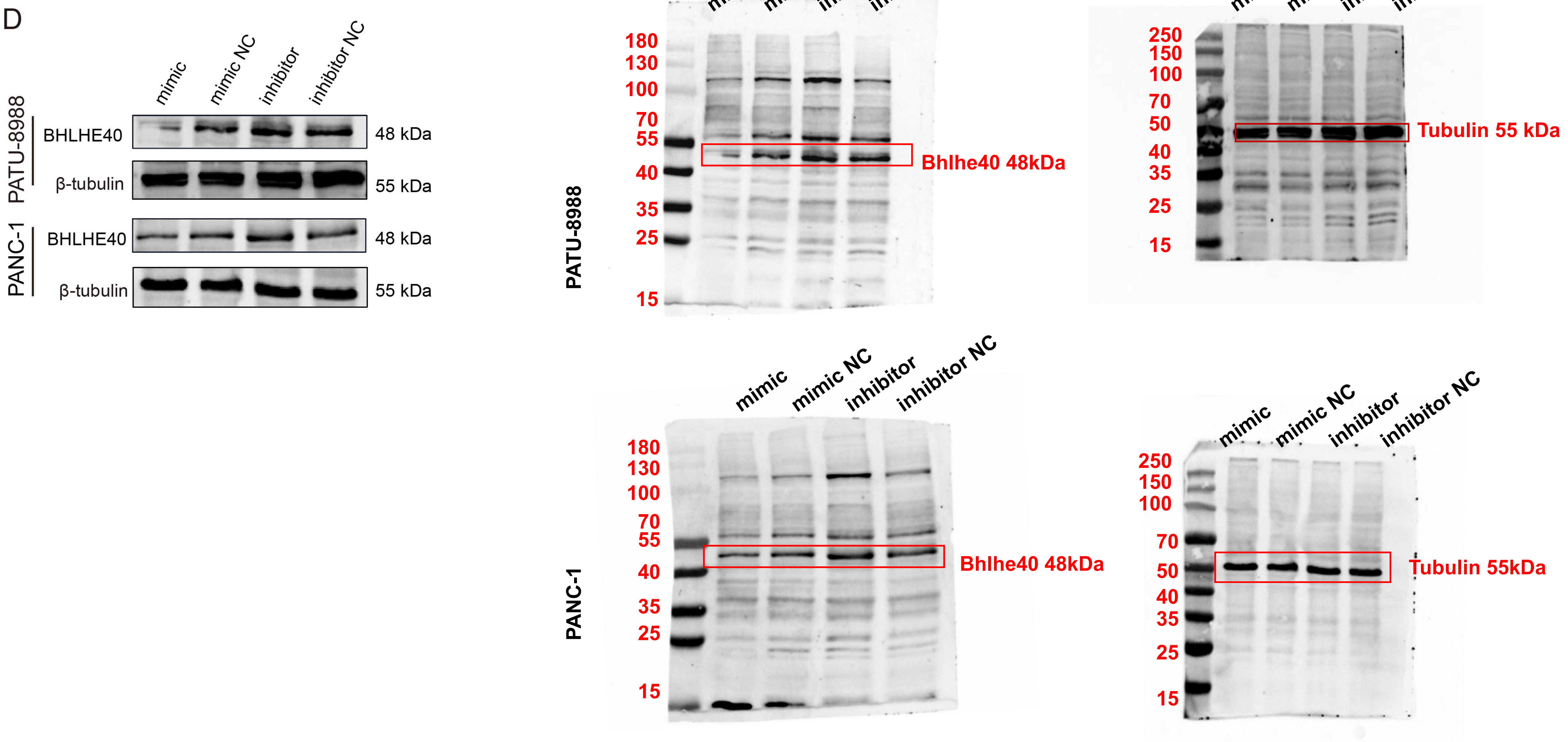
D
mimic
mimic NC
inhibitor
inhibitor NC
PATU-8988
BHLHE40
48 kDa
β-tubulin
55 kDa
PANC-1
BHLHE40
48 kDa
β-tubulin
55 kDa
PATU-8988
180
130
100
70
55
40
35
25
15
Bhlhe40 48kDa
250
150
100
70
50
40
35
25
15
Tubulin 55 kDa
PANC-1
Bhlhe40 48kDa
Tubulin 55kDa

Figure 11

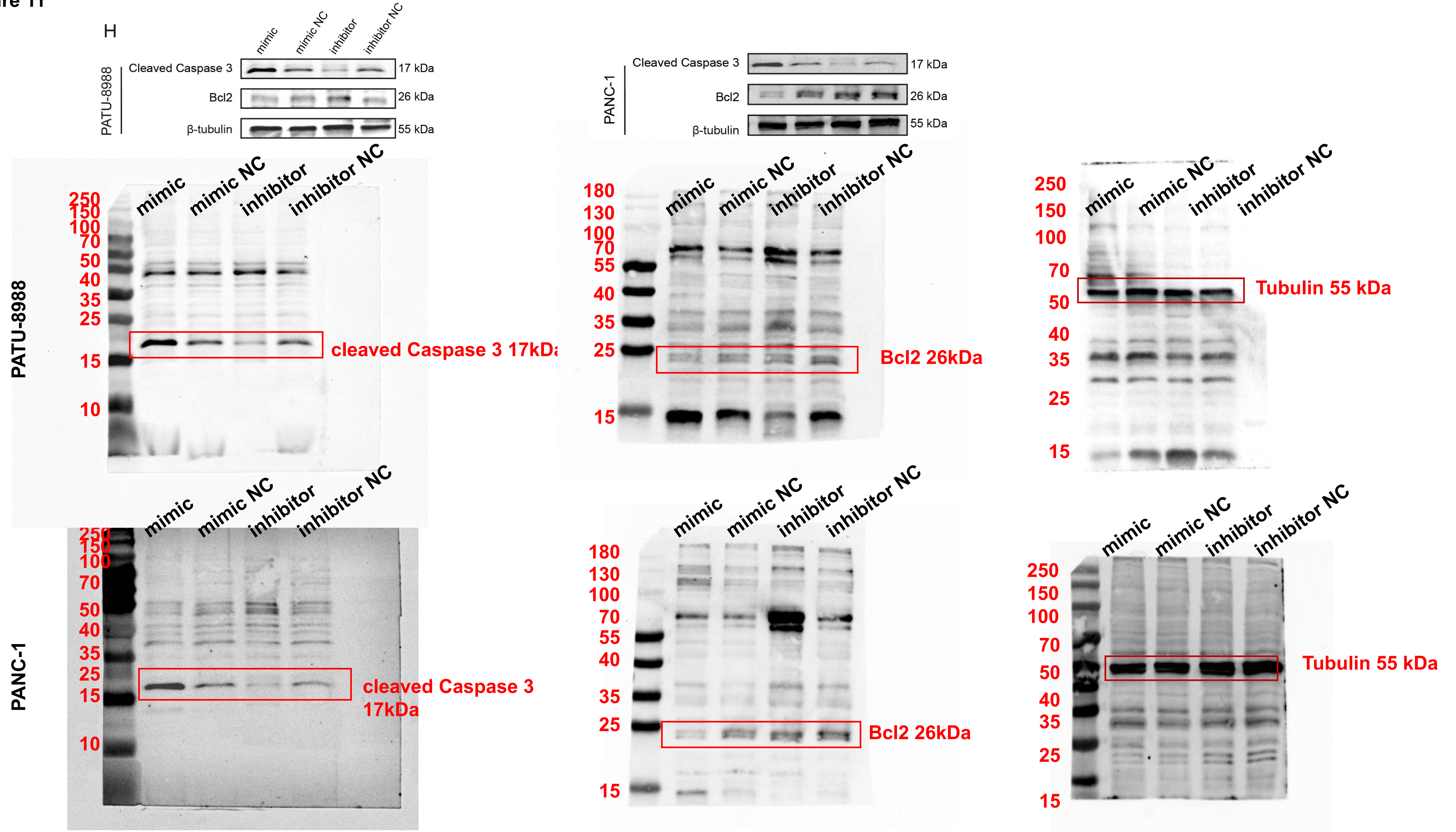

H
mimic
mimic NC
inhibitor
inhibitor NC
PATU-8988
Cleaved Caspase 3
17 kDa
Bcl2
26 kDa
β-tubulin
55 kDa
PANC-1
Cleaved Caspase 3
17 kDa
Bcl2
26 kDa
β-tubulin
55 kDa
PATU-8988
250
150
100
70
50
40
35
25
15
10
cleaved Caspase 3 17kDa
180
130
100
70
55
40
35
25
15
Bcl2 26kDa
250
150
100
70
50
40
35
25
15
Tubulin 55 kDa
PANC-1
250
150
100
70
50
40
35
25
15
10
cleaved Caspase 3
17kDa
180
130
100
70
55
40
35
25
15
Bcl2 26kDa
250
150
100
70
50
40
35
25
15
Tubulin 55 kDa

Figure 12

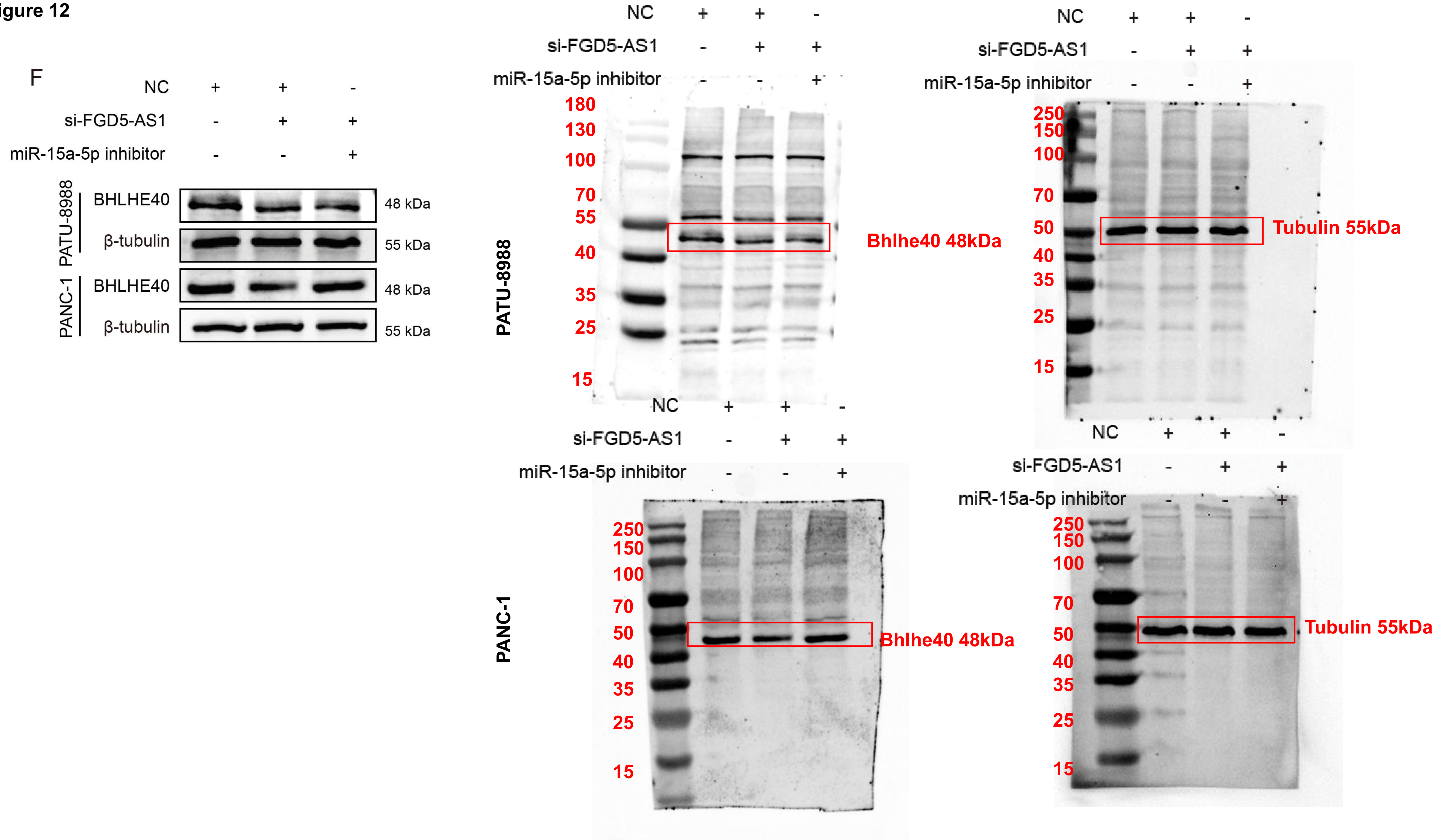
F
NC + + -
si-FGD5-AS1 - + +
miR-15a-5p inhibitor - - +
PATU-8988
BHLHE40
48 kDa
β-tubulin
55 kDa
PANC-1
BHLHE40
48 kDa
β-tubulin
55 kDa
NC + + -
si-FGD5-AS1 - + +
miR-15a-5p inhibitor - - +
180
130
100
70
55
40
35
25
15
Bhlhe40 48kDa
PATU-8988
NC + + -
si-FGD5-AS1 - + +
miR-15a-5p inhibitor - - +
250
150
100
70
50
40
35
25
15
Tubulin 55kDa
NC + + -
si-FGD5-AS1 - + +
miR-15a-5p inhibitor - - +
250
150
100
70
50
40
35
25
15
Bhlhe40 48kDa
PANC-1
NC + + -
si-FGD5-AS1 - + +
miR-15a-5p inhibitor - - +
250
150
100
70
50
40
35
25
15
Tubulin 55kDa

Figure 12

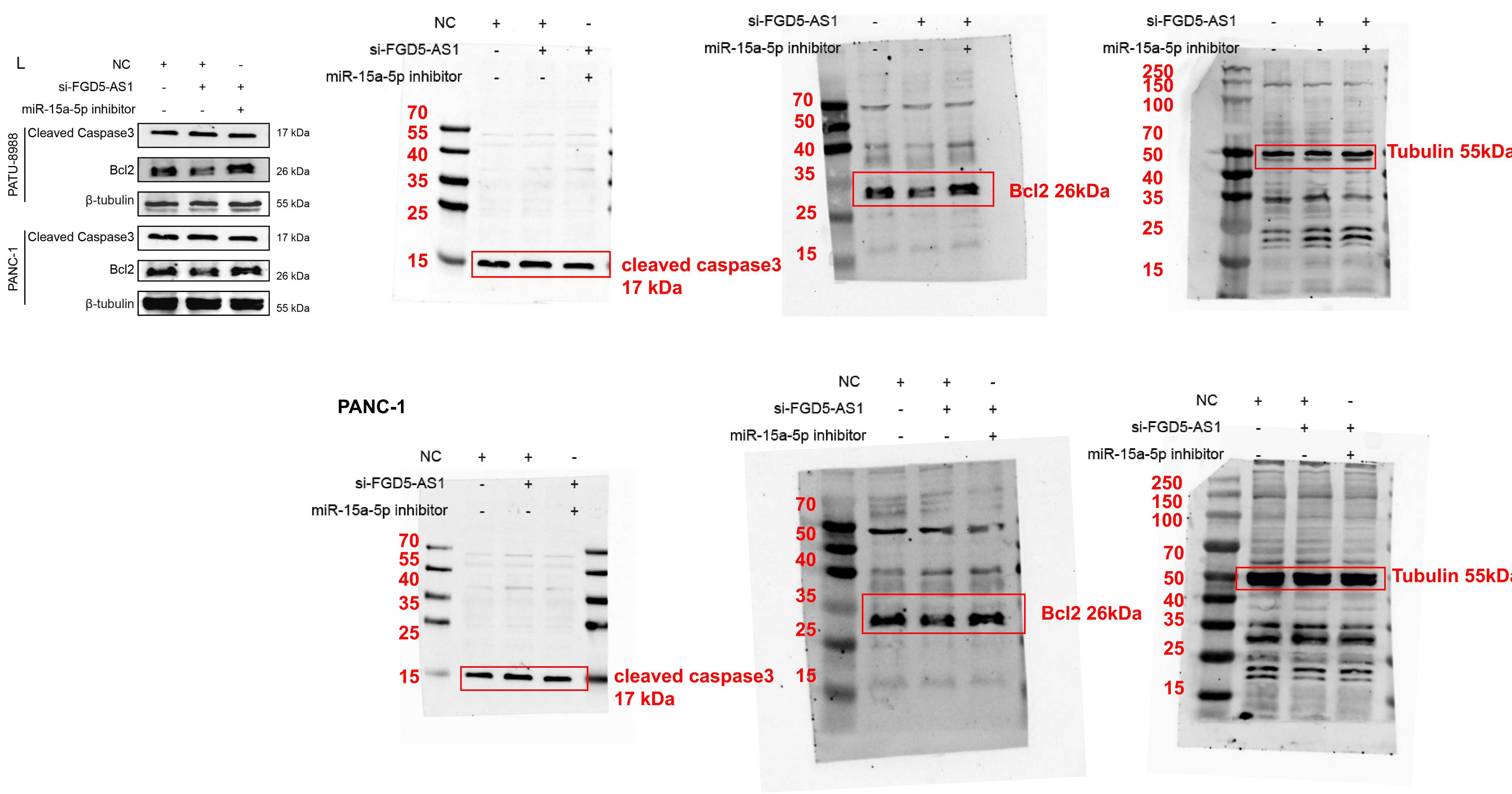
PATU-8988
PANC-1
L
NC
si-FGD5-AS1
miR-15a-5p inhibitor
Cleaved Caspase3
Bcl2
β-tubulin
17 kDa
26 kDa
55 kDa
cleaved caspase3 17 kDa
Bcl2 26kDa
Tubulin 55kDa
250
150
100
70
55
50
40
35
25
15
